# Immune microenvironment in hepatocellular carcinoma: from pathogenesis to immunotherapy

**DOI:** 10.1038/s41423-025-01308-4

**Published:** 2025-06-11

**Authors:** Deniz Seyhan, Manon Allaire, Yaojie Fu, Filomena Conti, Xin Wei Wang, Bin Gao, Fouad Lafdil

**Affiliations:** 1 Doctoral School of “Physiologie, Physiopathologie & Thérapeutique”, Sorbonne Université, Paris, France; 2 Laboratory of Liver Diseases, National Institute on Alcohol Abuse and Alcoholism, National Institutes of Health, Rockville, MD USA; 3 Pitié Salpêtrière University Hospital, Paris, France; 4 Genomic Instability, Metabolism, Immunity and Liver Tumorigenesis Laboratory, Equipe Labellisée LIGUE, Paris, France; 5 Centre de Recherche des Cordeliers, Sorbonne Université, INSERM, Université de Paris, Paris, France; 6 Laboratory of Human Carcinogenesis, National Cancer Institute, NIH, Bethesda, MD USA; 7 Liver Cancer Program, Center for Cancer Research, National Cancer Institute, NIH, Bethesda, MD USA; 8 Université Paris-Est Créteil (UPEC), Créteil, France; 9 INSERM, U938, Centre de Recherche Saint-Antoine (CRSA), Paris, France; 10 Sorbonne University, Paris, France; 11 Institut Universitaire de France (IUF), Paris, France

**Keywords:** Hepatocellular carcinoma, Immune microenvironment, Immunotherapy., Cancer microenvironment, Immunosurveillance

## Abstract

Hepatocellular carcinoma (HCC) is an increasingly prevalent and deadly disease that is initiated by different etiological factors, such as alcohol-associated liver disease (ALD), metabolic dysfunction-associated steatohepatitis (MASH), viral hepatitis, and other hepatotoxic and hepatocarcinogenic agents. The tumor microenvironment (TME) of HCC is characterized by several different fibroblastic and immune cell types, all of which affect the initiation, progression and metastasis of this malignant cancer. This complex immune TME can be divided into an innate component that includes macrophages, neutrophils, dendritic cells, myeloid-derived suppressor cells, mucosal-associated invariant T cells, natural killer cells, natural killer T cells, and innate lymphoid cells, as well as an adaptive component that includes CD4^+^ T cells, CD8^+^ T cells, regulatory T cells, and B cells. In this review, we discuss the latest findings shedding light on the direct or indirect roles of these immune cells (and fibroblastic-like cells such as hepatic stellate cells) in the pathogenesis of HCC. Henceforth, further characterization of this heterogeneous TME is highly important for studying the progression of HCC and developing novel immunotherapeutic treatment options. In line with this, we also review novel groundbreaking experimental techniques and animal models aimed at specifically elucidating this complex TME and discuss emerging immune-based therapeutic strategies intended to treat HCC and predict the efficacy of these immunotherapies.

## Introduction

Liver cancer, an umbrella term grouped together with hepatocellular carcinoma (HCC), intrahepatic cholangiocarcinoma (iCCA), hepatoblastoma, and hepatic angiosarcoma, represents the third leading cause of cancer-related deaths in 2022, with ~758,000 deaths worldwide, a number that is projected to increase to ~1,420,000 by 2050 [1]. Importantly, the number of recorded cases in 2022, 866,000, is approximately equal to a 1:1 incidence-to-death ratio [1], highlighting the importance of drawing further attention to this highly malignant disease. HCC is the most common and the most researched form of primary liver cancer, accounting for 80% of all liver cancer cases [2]. In light of these data, several studies and trials have developed numerous systemic treatments ranging from chemotherapies to immunotherapies, as well as nonsystemic treatments such as surgical interventions, with the aim of combatting this global epidemiological burden [3]. Although these treatment options are effective in alleviating symptoms and improving the prognosis of patients to a certain degree, HCC is still a life-threatening disease for which further fundamental and clinical research is needed. Since HCC commonly arises from a chronically inflamed liver background [4], regardless of the underlying etiology, many researchers and organizations have focused for several decades on the immune profile and characteristics of the HCC microenvironment (HCC-ME).

Human HCC usually develops on a highly inflammatory cirrhotic background, with 1–4% of cirrhotic patients eventually developing HCC annually [5]. Liver cirrhosis can itself develop from various etiologies, such as alcohol-associated liver disease (ALD) caused by excessive alcohol consumption and metabolic dysfunction-associated steatotic liver disease (MASLD) caused by the general consumption of a Western diet, along with advanced forms of alcohol-associated steatohepatitis (ASH) and metabolic dysfunction-associated steatohepatitis (MASH), respectively [6, 7]. Other exposomic factors that may cause chronic liver inflammation without necessarily leading to cirrhosis are infections from hepatic viruses such as hepatitis B virus (HBV) and hepatitis C virus (HCV), as well as risk factors such as exposure to aflatoxins, tobacco smoking, and genetic background. Up to 5.6% of patients with ALD-related cirrhosis develop HCC annually, accounting for almost one-fifth of HCC-related deaths in 2019 [8]. This number is up to 2.6% among patients with MASH-related cirrhosis [9]. Although hepatic viral infections are the most prevalent etiology behind liver cancer incidence, global vaccination efforts for hepatitis B virus (HBV) and antiviral treatments against hepatitis C virus (HCV) have significantly decreased the prevalence of these infections in the developed world; meanwhile, global consumerism in developed countries is causing an increase in ALD and MASLD and, consequently, HCC [10]. The combination of persistent chronic inflammation and functional defects from cirrhosis results in the formation of dysplastic nodules, marking the beginning of hepatocarcinogenesis [11]. While iCCA arises from mutations within hepatic bile duct cells, HCC arises from preneoplastic mutations originating from the parenchyma, mainly hepatocytes. However, several iCCA subtypes can be induced by etiologies similar to those of HCC, such as viral hepatitis, nonhepatitis viral infections, and diabetes [12, 13], in addition to both malignancies showing similar molecular features [14]. Some studies also suggest that hepatocytes may be the origin of iCCA tumors, since the decision to initiate HCC or iCCA may be dictated by microenvironment regulation [15–17].

HCC is caused mainly by genetic and/or epigenetic alterations resulting from the accumulation of gene mutations, leading to abnormal signaling pathways and uncontrolled proliferative cell activity, which leads to considerable genomic and functional heterogeneity among or within each tumor [18–21]. The means by which newly formed tumors progress could be explained by the immunoediting concept, which is composed of three steps [22]. In the first step, called “the elimination step”, altered cells express surface tumor antigens, which are recognized and killed by infiltrating immune cells, including natural killer (NK) cells and CD8^+^ cytotoxic T lymphocytes (CTLs). In the second step, an equilibrium between the proliferation of tumor cells and their elimination is observed. In the third step, tumor cells develop different strategies allowing them to escape immune surveillance. They express several cell surface molecules named immune checkpoints, which interact with complementary molecules expressed on T cells and lead to their inactivation and/or apoptosis. In addition, tumor cells become less sensitive to the cytotoxic inflammatory factors released in the microenvironment. When tumors reach a critical size, angiogenesis is initiated and constitutes an essential step for providing all the nutrients and oxygen required for their growth [23].

However, HCC remains a complex malady and needs further fundamental research to develop cutting-edge treatment options such as novel immunotherapies. We propose here to summarize the latest findings in the field of liver cancer research and its microenvironment, including the immune cells involved in the initiation and progression steps of HCC. We also summarize novel tools and therapeutic strategies aimed at controlling the associated immune response responsible for HCC pathogenesis.

## Characterization of the HCC microenvironment

### Involvement of fibroblastic cells in promoting HCC development

#### Hepatic stellate cells (HSCs)

HSCs are among the primary instigators of precancerous fibrosis through the production of various extracellular matrix (ECM) components following their transdifferentiation into α-smooth muscle actin-expressing myofibroblasts [24–26], gradually turning the liver into a cirrhotic and inflamed phenotype that favors the development of HCC. This ECM overproduction phenotype is believed to normally be an endogenous response that aids in tissue repair after acute injury, resulting in an overwhelming chronic wound healing response [27]. This aberrant repair mechanism is the result of repetitive liver injury induced by several factors, such as alcohol, MASH, hepatic viral infections, gut-derived toxins, and excessive inflammation. In contrast to its advanced cirrhotic state, liver fibrosis is reversible [28, 29]. Henceforth, the role of HSC-mediated fibrosis in the initiation and progression of HCC must be further experimentally studied to develop preventative treatments and potentially halt the initiation of hepatocarcinogenesis.

HSCs are known to be activated through various different mechanisms and pathways after liver injury, ranging from cytokines and chemokines to oxidative stress and autophagy [25, 30]. Recently, Li et al. reported that fibroblast growth factor-12 (FGF12)-expressing proinflammatory macrophages activate quiescent HSCs through the monocyte chemoattractant protein-1 (MCP-1)/C-C motif chemokine receptor-2 (CCR2) axis [31]. Neutrophils also activate HSCs through the production of neutrophil extracellular traps (NETs) by indirectly upregulating the ATP production required for the increased metabolic needs of myofibroblasts [32], mirroring the role of autophagy in HSC activation [33]. Macrolipophagy, a specific form of lipid autophagy, is correlated with HSC activation in the context of MASH [34]. Acetyl-CoA carboxylase (ACC) is an essential factor in the TGF-β1-mediated activation of HSCs [35]. HCC cells were also found to activate HSCs through the exosomal delivery of Smoothened (SMO) directly to HSCs by HCC cells [36] because SMO is part of the Hedgehog signaling pathway, a known HSC activator and fibrotic factor. Interestingly, sinusoidal accumulation of bile acids also leads to the activation of HSCs via farnesoid X receptor (FXR) [37]. Mesenchymal stromal cells might also play a minor role in the activation of HSCs in the fibrotic liver [38].

Filliol et al. reported that nonactivated quiescent HSCs and cytokine-producing HSCs play a protective role in the context of HCC, as opposed to their activated myofibroblastic and tumor-promoting forms, further suggesting that HSC activation plays a considerable role in HCC formation and proliferation [39]. Notably, this same study revealed that myofibroblastic HSCs initiate HCC through collagen-dependent TAZ accumulation in hepatocytes and promote HCC through discoidin domain receptor-1 (DDR1) activation. A more direct involvement of HSCs in the progression of HCC was illustrated by a recent study in which activated HSCs secreted hexokinase 1 (HK1)-containing extracellular vesicles, which are used by HCC cells via increased glycolysis [40]. For example, some in vitro studies revealed that HSCs directly promote carcinogenic hallmarks such as angiogenesis and epithelial‒mesenchymal transition (EMT) [41, 42]; however, further in vivo studies are needed. Moreover, senescent activated HSCs were shown to play an important role in the transformation of steatotic hepatocytes into tumor cells in MASH murine models [43]. A study using diethylnitrosamine (DEN)/carbon tetrachloride (CCL_4_)-induced HCC transgenic mice demonstrated the role of the Cyclin E1/cyclin-dependent kinase-2 (CDK2) axis in promoting HCC [44]. Increased autophagy also results in the production of growth differentiation factor-15 (GDF15) by HSCs, which favors HCC development [45]. Furthermore, cirrhotic patients treated with sorafenib undergo HSC ferroptosis through epigenetic regulation [46], characterizing an additional HSC-dependent pathway that explains the efficiency of sorafenib in treating HCC. In addition to indirectly promoting hepatocarcinogenesis, some studies have highlighted the potential of HSCs to directly promote HCC progression through the secretion of regulatory proteins [45, 47, 48]. Interestingly, HSCs are also correlated with promoting HCC by creating genomic instability through histone lactylation [49]. HSCs and monocyte interactions may contribute to HCC progression by inducing protumorigenic and progressive features of tumor cells, such as cell migration and tumor sphere formation [50]. A recent study demonstrated the effectiveness of celastrol in CCl_4_-treated mice, in which activated HSCs underwent ferroptosis and partially rescued the induced fibrotic phenotype [51].

#### Cancer-associated fibroblasts (CAFs)

In addition to being the main source of myofibroblasts, quiescent HSCs are also the origin of ~85% of cancer-associated fibroblasts (CAFs) in HCC-ME [52, 53]. CAFs can also be derived from other mesenchymal cells, as well as epithelial and endothelial cells, resulting in their highly heterogeneous and multifaceted phenotype in the context of HCC [27]. Owing to their presence in the TME, CAFs interact with many immune cells. Notably, CAFs can directly induce the secretion of chemokines such as CXCL6 by cancer cells to aid in the recruitment of TANs within the TME [54]. CAF-specific galectin-1 (Gal-1) targeting decreases HCC progression in vivo [55]. Specific subsets of CAFs were found to recruit CD33^+^ myeloid-derived suppressor cells (MDSCs) in a macrophage migration inhibitory factor (MIF)-dependent manner [56]. Tong et al. recently reported that tumor cells can induce the differentiation of HSCs into CAFs through the selective phosphoprotein-1 (SPP1)/CD44 axis, resulting in a positive feedback loop in the context of HCC progression [57]. Although ECM overproduction favors carcinogenesis, collagen type I-producing CAFs have been shown to inhibit tumor promotion due to increased TME stiffness [58], most likely by slowing the expansion of cancer cells. However, the same logic can be applied to the migration of oncolytic immune cells into the tumor site [59, 60], highlighting the need to further characterize this dual effect of CAF-induced ECM stiffness. To further underscore this confounding effect, another study revealed that this stiffness phenotype also activated HSCs and led to HCC progression [61]. The use of nanocarriers to specifically target HSCs and CAFs in vivo also has therapeutic potential for reducing fibrosis and even attenuating HCC expansion [62, 63].

### Impact of the gut‒liver axis on HCC development

The liver is anatomically and functionally linked to the gut through the portal circulation, rendering it continuously exposed to gut-derived microbial components. Disruption of the intestinal epithelial barrier—often described as a “leaky gut”—facilitates the translocation of bacterial products such as lipopolysaccharides (LPS), bacterial DNA, and metabolites into the liver. This microbial translocation activates hepatic immune cells through pattern recognition receptors such as TLRs, triggering chronic inflammation and contributing to fibrosis, a key step in hepatocarcinogenesis. The interplay between microbial signals and hepatic immune responses represents a critical component in the pathogenesis of HCC, particularly in the context of chronic liver injury and metabolic liver diseases [9, 64–66]. For example, NLRP6, a key regulator of the composition of the gut microbiota, has been shown to be involved in promoting the growth of beneficial bacteria within the gut, such as *Akkermansia muciniphila*. Indeed, experiments conducted in an NLRP6-deficient transgenic mouse model revealed that intestinal dysbiosis and loss of *A. muciniphila* disrupted epithelial barrier integrity and promoted hepatic monocytic-MDSC (M-MDSC) expansion in a TLR4-dependent manner, suppressing CD8^+^ T-cell activity and accelerating HCC progression—an effect reversible by antibiotic treatment or *A. muciniphila* reconstitution [67].

Emerging evidence has shown that alterations in the composition of the gut microbiota—often referred to as dysbiosis—are associated with the progression of chronic liver disease and the development of HCC. In particular, a study demonstrated that a high-fat/high-cholesterol (HFHC) diet induces distinct microbial dysbiosis associated with altered production of ROS and microbial metabolites, including increased taurocholic acid (TCA) and reduced levels of 3-indolepropionic acid (IPA), which promotes the transition from MASLD to HCC [68]. Furthermore, gut-derived D-lactate has been shown to switch TAMs from an immunosuppressive M2 phenotype to a proinflammatory M1 phenotype in HCC to promote antitumor immunity [69]. In mice subjected to the DEN-CCl_4_ model, reduced gut microbial diversity and lower acetate levels were associated with increased IL-17A-producing group 3 innate lymphoid cells (ILC3s) and greater tumor burdens, whereas restoring acetate through fecal microbiota transplantation or supplementation suppressed IL-17A production via histone deacetylase inhibition and Sox13 downregulation, thereby limiting fibrosis and tumor progression [70]. The role of gut-derived acetate was further studied in a MASLD-HCC mouse model, in which *Bifidobacterium pseudolongum* was markedly depleted, and its metabolite, acetate, was found to exert protective effects by restoring gut barrier integrity, suppressing LPS translocation, and inhibiting the pro-oncogenic IL-6/JAK1/STAT3 pathway via GPR43 signaling in hepatocytes, ultimately reducing tumor burden [71]. In both murine and human MASH, intestinal B cells—despite acting independently of the gut microbiota—promote hepatic inflammation and fibrosis by activating CD8^+^ T cells and driving IgA-FcR signaling in macrophages, underscoring their dual role in immune activation and fibrogenesis relevant to HCC progression [72]. A Mendelian randomization study combined with case‒control validation identified protective associations between liver cancer risk and specific gut microbes, notably *Ruminococcaceae*, *Porphyromonadaceae*, and *Bacteroidetes*, whose reduced abundance in patients highlights their potential role in liver cancer prevention [73]. The reduced abundance of *Bacteroides thetaiotaomicron* in recurrent HCC patients was linked to diminished acetic acid-mediated polarization of M1 macrophages and CD8^+^ T-cell activation, with mechanistic studies showing that this effect involves histone acetylation-dependent upregulation of ACC1 and is critical for maintaining an antitumor immune microenvironment [74]. Emerging research highlights a bidirectional relationship between host genetics and the gut microbiome, where genetic variants shape microbial composition, and gut-derived metabolites influence host epigenetic regulation, including DNA methylation and histone modification, contributing to MASLD progression and HCC risk while also offering therapeutic and biomarker potential [75].

Recent studies have begun to link the gut microbiota composition with the efficacy of immune checkpoint inhibitors (ICIs) in patients with HCC, suggesting that certain microbial profiles may predict therapeutic outcomes [76, 77]. Certain gut microbes, such as *Bacteroides fragilis* and butyrate-producing bacteria, have been shown to enhance antitumor immune responses, whereas dysbiosis involving pathogenic species is linked to poor outcomes, underscoring the importance of the microbiota in shaping immunotherapy efficacy [78]. The protective role of butyrate is underscored by the finding that its systemic reduction is correlated with HCC patients and that it enhances anticancer therapy by disrupting calcium homeostasis while synergizing with sorafenib to suppress tumor growth and metastasis, as demonstrated by a novel nanoparticle-based codelivery strategy [79]. However, another study revealed that a distinct gut microbiome marked by increased short-chain fatty acid (SCFA)-producing bacteria, such as *Bacteroides* and *Veillonella*, in MASLD-HCC patients was associated with elevated serum and fecal butyrate levels, which in turn promoted an immunosuppressive environment by expanding IL-10^+^ T_regs_ and reducing cytotoxic CD8^+^ T-cell function, highlighting the antagonistic effects of gut-derived butyrate on HCC progression [80]. In patients with unresectable HCC undergoing immunotherapy, enrichment of *Lachnoclostridium* and associated bile acids such as ursodeoxycholic acid correlated with improved survival and objective response, whereas *Prevotella 9* abundance was linked to poor outcomes, highlighting the predictive value of the fecal microbiota and bile acid profiles in ICI efficacy [81]. Multiomics profiling of ICI-treated HCC patients revealed that those with durable clinical benefit presented greater gut microbiota beta diversity and distinct bacterial–metabolite networks, with species such as *Senegalimassilia anaerobia* and metabolites such as galanthaminone emerging as predictive markers of immunotherapy response and overall survival [82]. Microbiome diversity has also been shown to influence radiotherapy outcomes in HCC, with enriched genera such as *Faecalibacterium* linked to improved responses through cGAS-STING-IFN-I pathway activation, whereas dysbiosis and loss of microbial signals such as cyclic di-AMP impair immune-mediated tumor control, as demonstrated in both patients and mouse models [83]. Loss of AKR1D1 promotes gut microbiota-driven accumulation of isolithocholic acid via *Bacteroides ovatus*, impairing NK cell cytotoxicity through p-CREB1 signaling and accelerating HCC progression, an effect that can be therapeutically targeted to increase anti-PD1 efficacy [84]. In conclusion, the gut microbiota intricately shapes both innate and adaptive immune responses in HCC by influencing key immune cell populations, such as T_regs_, MDSCs, CD8^+^ T cells, and DCs, and increasing evidence suggests that modulating the microbiome through strategies such as fecal microbiota transplantation, probiotics, antibiotics, dietary interventions, or nanodelivery systems may help control HCC development and improve clinical outcomes, such as by enhancing the efficacy of ICIs [85, 86].

### Impact of innate immune cells on HCC development

**Fig. 1:**
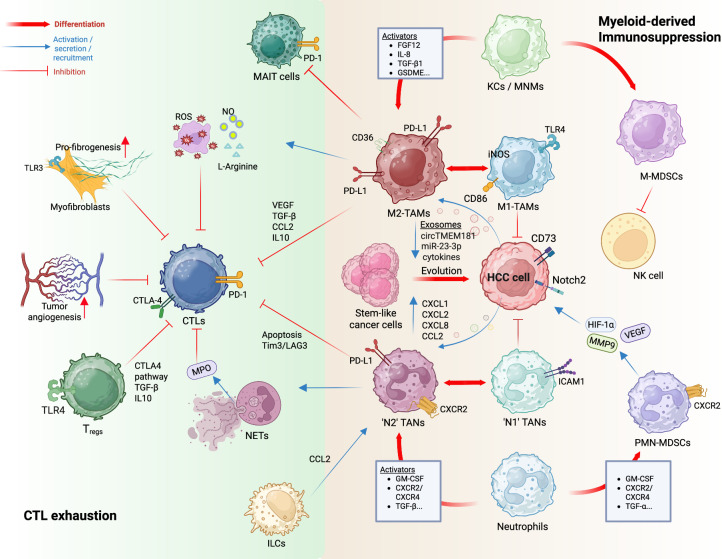
Impact of myeloid cell-mediated immunosuppression and cytotoxic T-cell exhaustion within the hepatocellular carcinoma immune microenvironment. One of the prominent members of the immune TME is myeloid cells, which mainly consist of TAMs derived from resident KCs or infiltrating MNMs through the action of various novel molecules and cytokines. TAMs secrete a wide variety of proinflammatory cytokines, such as IL-1β, IL-6 and TNF-α, that are involved in the initiation and progression of HCC. TAMs also act on all steps of HCC growth via various newly discovered mediators and molecules. TANs constitute another major type of myeloid cell that is commonly observed in the HCC TME and is differentiated from neutrophils through various chemokines. TANs also produce proinflammatory cytokines such as TNF-α and secrete NETs that favor HCC growth. In addition to TAMs and TANs, macrophages and neutrophils can also differentiate into M-MDSCs and PMN-MDSCs, respectively, which also play tumor-promoting and immunosuppressive roles within the TME. Owing to their inherent cytotoxic and antitumor role, CTLs are prone to inhibition and exhaustion mediated by the surrounding TME through various complex and heterogeneous mechanisms, ranging from extracellular molecules to interactions with other immune cell types. TME tumor microenvironment, HCC hepatocellular carcinoma, TAMs tumor-associated macrophages, MNMs monocyte-derived macrophages, KCs Kupffer cells, CTLs cytotoxic T lymphocytes, M-MDSCs monocytic myeloid-derived suppressor cells, PMN-MDSCs polymorphonuclear myeloid-derived suppressor cells, NK natural killer, TANs tumor-associated neutrophils, NETs neutrophil extracellular traps, IL interleukin, TNF tumor necrosis factor, TLR toll-like receptor, ROS reactive oxygen species, NO nitric oxide, VEGF vascular endothelial growth factor, TGF transformant growth factor, CCL C-C motif ligand, CTLA cytotoxic T lymphocyte-derived suppressor protein, T_regs_ regulatory T cells, MPO myeloperoxidase, TIM = T-cell immunoglobulin, LAG lymphocyte activation gene, PD-1 programmed cell death, PD-L1 programmed cell death ligand, 3-HAA = 3-hydroxyanthralinic acid, FGF fibroblast growth factor, CXCL C-X-C motif ligand, TMEM transmembrane protein, GM-CSF granulocyte‒macrophage colony-stimulating factor, GSDME gasdermin E, iNOS inducible nitric oxide synthase, Notch neurogenic locus notch homolog protein, ICAM intercellular adhesion molecule

**Fig. 2:**
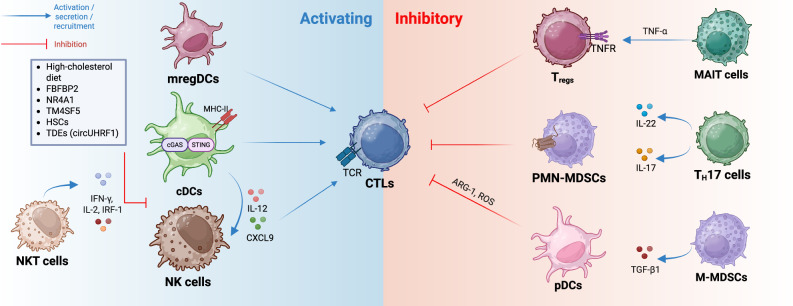
Activating and inhibitory effects of other innate and adaptive immune cell types within the hepatocellular carcinoma microenvironment. In contrast to cDCs and mregDCs, anti-inflammatory pDCs can result in the exhaustion phenotype of CTLs through various mechanisms. PMN-MDSCs and T_regs_ also have direct inhibitory effects on the cytotoxic function of CTLs. T_H_17 cells, M-MDSCs and MAIT cells are also indirectly involved in the dysfunction phenotype of CTLs. NK cells are known antitumor protagonists through their cytotoxic activity; hence, HCC-ME inhibits the cytotoxic properties of NK cells. NKT cells are another type of lymphoid cell that exhibit antitumor functions. TME tumor microenvironment, HCC hepatocellular carcinoma, CTLs cytotoxic T lymphocytes, cDCs conventional dendritic cells, pDCs plasmacytoid dendritic cells, mregDCs mature DCs enriched in immunoregulatory molecules, M-MDSCs monocytic myeloid-derived suppressor cells, PMN-MDSCs polymorphonuclear myeloid-derived suppressor cells, NK natural killer, NKT cells natural killer T, MAIT mucosal-associated invariant T, IL interleukin, TGF transformant growth factor, IFN-γ interferon-γ, TNF tumor necrosis factor, TNFR TNF receptor, HSCs hepatic stellate cells, IRF interferon regulatory factor, NR4A1 nuclear stellate cells-4A1, TM4SF1 transmembrane-4 L six family member-1, TDEs tumor-derived exosomes, circUHRF1 circular ubiquitin-like with PHD and ring finger domain-1, CXCL C-X-C motif ligand, cGAS cyclic GMP-AMP, STING stimulator of interferon genes, TCR T-cell receptor, ARG arginase, ROS reactive oxygen species

The liver is enriched with a unique population of resident immune cells that play a central role in maintaining immune homeostasis and orchestrating responses to hepatic injury and malignancy. In the context of HCC, this immunological niche becomes significantly altered, with resident immune cells contributing to both tumor-promoting inflammation and antitumor surveillance. Key players such as resident myeloid cells (such as Kupffer cells (KCs) and dendritic cells), liver-resident NK cells, and tissue-resident T cells adapt to the TME, as do resident ILC3s, where their phenotypic and functional plasticity profoundly influences HCC progression and immune evasion (Figs. 1, 2).

#### Macrophages

Macrophages are generally found in two forms in the liver: infiltrated monocyte-derived macrophages and liver-resident macrophages named KCs, with the latter representing approximately one-third of nonparenchymal cells in the human liver [87–89]. More intricately, macrophages are classified according to their polarization into an M1-like phenotype or an M2-like phenotype, discerned from each other by their proinflammatory and anti-inflammatory makeup, respectively [90]. Differentiation into these distinct polarized states is achieved by several cytokines and/or exogenous factors [91], such as LPS, tumor necrosis factor (TNF) and IFN-γ for M1-like macrophages and IL-4, IL-13 and TGF-β for M2-like macrophages. While TAMs are widely referred to as anti-inflammatory macrophages present in the TME, some aspects of the current scientific literature may interchangeably use M2-like macrophages and TAMs; however, TAMs display the phenotypic traits of both M1-like and M2-like macrophages [92]. This is probably the result of the heterogeneous origin of TAMs within the TME, in addition to the dynamic phenotype switching known to be exhibited by macrophages. In general, most TAMs exhibit a Ly6C^low^ CD11b^low^ F4/80^high^ phenotype after their transformation into an M2-like phenotype in mice [92].

The most studied impact of macrophages on the progression of HCC is their role in immune evasion/escape, notably through their malicious use of ICIs such as the PD-L1/PD-1 axis to induce immunosuppression, mainly toward CD8^+^ T cells. For example, the RNA demethylase AlkB homolog-5 (ALKBH5) upregulates MAP3K8 via the demethylation of its mRNA within HCC cells to indirectly lead to the expression of IL-8 and recruitment of PD-L1^+^ macrophages [93]. In addition to the recruitment of PD-L1^+^ macrophages, some factors can promote the expression of PD-L1 on the surface of TAMs, such as TGF-β1-mediated SRY-related HMG-box 18 (SOX18) overexpression, which is able to upregulate CXCL12 and PD-L1 expression within TAMs [94]. Miz1 was recently characterized as an important regulator of HCC progression within hepatocytes, where its absence results in the activation of anti-inflammatory TAMs due to a lack of metadherin-MTDH-mediated (a coactivator) NF-κB inhibition [95]. Interestingly, macrophages enriched with tumor-derived lipid droplets can recruit T_regs_ via the CCL20‒CCR6 axis [96]. A recent study characterized another subset of lipid-enriched TAMs that are fatty-acid binding protein-5 (FABP5)^+^, with a highly immunosuppressive phenotype [97]. In anti-PD-1 therapy-resistant patients, tumor-derived exosomes (TDEs) containing circTMEM181 are internalized by TAMs to indirectly upregulate the expression of CD39 [98]. CD39, in turn, works in tandem with HCC cell-expressed CD73 to convert extracellular ATP into adenosine, a known actor in T-cell exhaustion [98]. CSF1R^+^ TAMs were recently characterized for their ability to directly cause mucosal-associated invariant T (MAIT) cell dysfunction via the PD-L1/PD-1 axis [99]. Subsequent PD-L1 immunotherapy led to the infiltration and increased cytotoxicity of MAIT cells within HCC tumor sites [99]. Exosomal miR-23a-3p released by M2-like macrophages leads to HCC metastasis through increased angiogenesis and EMT, as reported by in vitro experiments in which HCC cell lines treated with these exosomes presented increased migratory and invasive properties [100]. Their involvement in microvascular invasion was also recently documented via intricate single-cell RNA sequencing analyses [101]. The ferroptotic pathway has recently been described in detail as a modulator of the phenotypic switching phenomenon by favoring TAM differentiation toward an M1-like phenotype [102, 103]. This protective natural event can be decreased, for example, by the upregulation of transmembrane protein-147 (TMEM147) through signal transducer and activator of transcription-2 (STAT2), leading to the progression of HCC [104]. Xanthine oxidoreductase was also discovered to be involved in the proinflammatory polarization of TAMs [105]. Furthermore, gut-derived metabolites were also shown to switch macrophages from the M2 phenotype to the M1 phenotype [69]. Serine/arginine-rich splicing factor-10 (SRSF10)-induced lactate overproduction within tumor cells was also found to be involved in M1-to-M2 switching [106]. Lactate found in the TME can further inhibit zinc finger and homeobox protein-1 (ZHX2), a molecule that regulates the M2-like polarization of TAMs [107]. Tumor cells can secrete other molecules that lead to M2-like phenotype switching, such as CSF1, a molecule that is transcriptionally regulated by ZFP64 within HCC cells [108]. Interesting phenotypic switching of macrophages was recently reported, in which fetal-like (FOLR2^+^) macrophages drove immunosuppression in various cross-species of tissues [109]. Patients who underwent transarterial chemoembolization (TACE) were found to harbor important amounts of TREM2^+^ macrophages that potentially play a role in the high recurrence rate of HCC after TACE [110]. Another subset of TAMs that expresses glycogen synthase kinase-3β (GSK3β) has been characterized as an anti-PD-1 therapy resistance marker [111]. Liu et al. characterized the presence of a “tumor immune barrier” consisting of CAFs and secreted phosphoprotein (SPP1)^+^ macrophages that seemingly limits the infiltration of cytotoxic T cells into the tumor and is linked with resistance to immunotherapy [112]. In parallel with this discovery, Ning et al. characterized a similar phenomenon, wherein tumors were protected from cytotoxic T cells by the formation of a macrophage-coated tumor cluster enveloping the tumor site [113]. Macrophages are also involved in the initiation of HCC by promoting fibrosis via CCR2-dependent transdifferentiation of HSCs [31]. This is further underscored by their positive interaction with stem-like tumor cells, resulting in posttherapy HCC recurrence, as demonstrated in mouse models [114]. Furthermore, gasdermin E, a pyroptosis factor generally expressed by TAMs within immune cells, activates the PI3K‒AKT pathway to promote M2-like polarization, and its suppression results in a decrease in this polarization [115]. Additionally, sirtuin-5 (SIRT5) is characterized as a regulator of bile acid production that consequently leads to decreased immunosuppression, since bile acids are known to promote M2-like macrophage polarization [116]. The overexpression of ETS translocation variant-4 (ETV4) in HCC cells leads to the activation of PD-L1 and CCL2, which consequently results in the recruitment of TAMs and MDSCs to the tumor site [117]. Tumor-derived ectosomal pyruvate kinase M2 (PKM2) promotes HCC by inducing the differentiation of monocytes into M2-like macrophages [118]. TAM-specific ferroptosis induction resulted in significant improvements in PD-L1 therapy [119]. Bufalin, a cardiotonic steroid derivative isolated from venomous Chinese toads, allows for the polarization of M2-like macrophages to M1-like macrophages, highlighting its potential therapeutic use [120]. Finally, Li et al. developed a cutting-edge technique termed “macrophage hitchhiking”, in which macrophages are used to deliver kinase inhibitors directly into the tumor site, with great success in various HCC animal models [121].

The IL-1 family of cytokines contains 11 members, including the highly inflammatory cytokines IL-1β and IL-1α. For example, macrophages are known inducers of IL-1β production and secretion. Gao et al. demonstrated that blocking autophagy resulted in the release of IL-1β through the inflammasome complex inside macrophages to contribute to HCC progression [122]. Furthermore, vimentin^high^ macrophages increase the release of IL-1β from T_regs_ to potentiate immunosuppression [123]. IL-1β can, in turn, indirectly upregulate the expression of protumor factors such as PD-L1 and CSF1 to promote the metastasis of HCC [124]. This metastatic feature of IL-1β was further characterized by the upregulation of IL-1β via CAF-secreted CXCL11, resulting in increased migratory properties of HCC cells, as demonstrated in vitro and in vivo [125]. Moreover, IL-1β can assist in HCC metastasis by overexpressing homeobox C10 (HOXC10), which itself acts on downstream metastatic molecular factors such as 3-phosphoinositide-dependent protein kinase-1 and vasodilator-stimulated phosphoprotein [126]. Additionally, a derivative of 18β-glycyrrhetinic acid, which is naturally found in liquorice, was characterized to act as an inhibitor of the IL-1β pathway to reverse its detrimental effects on HCC growth [127]. 3-Hydroxyanthralinic acid (3-HAA) was recently characterized as an anti-inflammatory factor because of its ability to inhibit the impact of several cytokines, such as TNF and IL-6, within (but not limited to) macrophages [128]. A recent study mechanistically characterized the role of Schlaffen-11 (SLFN11) in inhibiting the transcription of CCL2 and thus disallowing the M2-like polarization of monocytes [129].

#### Dendritic cells (DCs)

Conventional DCs (cDCs = myeloid DCs = mDCs) are antigen-presenting cells (APCs) that bridge innate and adaptive immunity by initiating T-cell responses through the presentation of antigens and costimulatory signals. cDCs can be subdivided into cDC1s and cDC2s according to their phenotypic traits, with cDC1s stimulating CD8^+^ T cells for a cytotoxic response and cDC2s stimulating CD4^+^ T cells. Another type of DC is composed of plasmacytoid DCs (pDCs), which are involved in IFN-1-mediated antiviral immunity. The increased presence of infiltrated cDCs within HCC tumors is generally considered a positive marker in patients, including immunotherapy responders [130, 131]. However, some specific immunosuppressive DC subsets, such as CD103^+^ PD-L1^+^ DCs, are observed in the peritumoral region of relapsed patients who have undergone surgical resection [132]. Furthermore, single-cell transcriptomic analysis of early-relapse patients revealed elevated numbers of DCs within the tumor site, with a likely dysfunctional phenotype likely due to competitive inhibition of their costimulatory signal by cancer cells [133]. On the other hand, pDCs are increasingly correlated with poor HCC prognosis, as they effectively recruit T_regs_ and inhibit cytotoxic CD8^+^ T cells after being recruited to the TME via the action of tumor-derived extracellular adenosine (eADO) [134]. pDCs also indirectly recruit MDSCs by secreting IFN-α on hepatocytes, which in turn recruit MDSCs via the CX3CL1-CX3CR1 axis to further elicit immunosuppression [135]. Moreover, mregDCs are a specific DC subgroup that expresses high amounts of *SLC7A11*, and the KO of this gene combined with radiofrequency ablation (RFA) therapy resulted in increased tumor regression in a subcutaneous tumor model compared with RFA monotherapy [136]. Magen et al. characterized the action of specific subsets of mregDCs and CD4^+^ T cells in potentiating the maturation and stimulation of CD8^+^ T cells in HCC patients who respond to PD-1 treatment [137]. Furthermore, blocking CD47 results in CD103^+^ DCs regaining their effector functions and recruiting NK cells via IL-12 and CXCL9 to combat HCC in an orthotopic tumor model [138]. Highly hypoxic tumor regions that are subject to immunosuppression are rich in HLA-DR^lo^ cDC2s, along with other anti-inflammatory immune cell types, such as T_regs_ and M2-like macrophages [139]. The cGAS-STING pathway, an innate immune defense pathway that functions via the recognition of dsDNA by cGAS and leads to the transcription of interferons by STING, is known to be heavily involved in HCC and other liver diseases [140, 141]. For example, PARP1 degradation within cancer cells leads to the generation of dsDNA that is recognized by the cGAS-STING pathway inside DCs and leads to the activation of antitumor adaptive responses [142]. Accordingly, the repair of dsDNA that is targeted by this same pathway consequently leads to immunosuppression due to a lack of DC activity, such as via the RECQL4-mediated dsDNA repair mechanism [143]. The targeted nanovaccine-induced pyroptosis of HCC cells was also observed to activate the cGAS‒STING pathway, which ultimately leads to DC maturation [144]. cDCs that have defective effector functions can be rescued by intratumoral injection of nanodrugs consisting of tumor-associated antigens that present enhanced internalization properties by DCs [145]. Another study further revealed the potential of salvaging this defective phenotype, in which they reported improved APC function in cDC1s following combined anti-PD-1 and anti-CXCR4 therapy [146]. The use of DC-derived exosomes as nanovaccine carriers also showed great promise when Zuo et al. demonstrated successful tumor eradication in an orthotopic HCC mouse model [147]. DC-derived nanovesicles containing anti-ICIs also look promising [148]. A different form of DC-based vaccine therapy with great effects has been tested in human patients and is correlated with an improved T-cell response and a low recurrence rate [149].

#### Neutrophils

Neutrophils are the first responders of the innate immune system, known for combating infections and tissue damage, which is facilitated by their main effector functions of degranulation and the production of proinflammatory cytokines. However, they, especially tumor-associated neutrophils (TANs), have been correlated with HCC for many decades [150, 151], including HCC recurrence following tumor resection [152]. Notably, the neutrophil-to-lymphocyte ratio (NLR) is widely accepted as a prognostic indicator in HCC [153, 154]. Like macrophages, neutrophils have two main polarized states, termed N1-like and N2-like neutrophils, with the N2-like state associated with TANs due to their protumorigenic and immunosuppressive phenotype. Owing to their heterogeneity and dynamic phenotypic switching ability, classifying them according only to this simple nomenclature would be a mistake [151]. Classically, neutrophils and TANs mainly express CXCR1, CXCR2, CXCR4, G-CSF, and CCR5, as well as several other chemokines and receptors, allowing their recruitment into infected sites or into the HCC-ME [155].

In the scientific literature, neutrophils have been shown to be involved in each step of HCC, ranging from hepatocarcinogenesis to metastasis, through direct and/or indirect actions in HCC-ME. For example, Teo et al. reported that a specific subset of TANs can promote tumor stemness, assist in immune escape and even promote metastasis in MASH-HCC [156]. Another study revealed their ability to promote mesenchymal transition via the secretion of TNF-α [157]. Neutrophils can produce chromatin-rich dense fibers—called neutrophil extracellular traps (NETs)—that are primarily used as an innate antibacterial defense mechanism. However, they have been found to be correlated with HCC progression [158, 159] and metastasis [160] in recent years. Furthermore, HBV-infected HCC cells can secrete S100A9, which indirectly increases ROS production within TANs and leads to the secretion of protumoral NETs, creating a malignant amplification loop [161]. HCC development can be further aided by NETs through their capacity to differentiate naïve CD4^+^ T cells into T_regs_ via TLR4 in the context of MASH-HCC [162]. NETs are also involved in MASH-HCC, in which diet- or chemically induced fibrotic MASH mice are correlated with increased NET production and are able to activate quiescent HSCs via TLR3 signaling, hence creating a milieu that potentially favors the initiation of HCC [32]. In addition, NETs can promote the infiltration of T_regs_ into the TME by inducing the surface expression of CD73 on tumor cells via Notch2 signaling, thus further increasing the immunosuppressive milieu of HCC [163]. NETs are also secreted in response to a collagen-rich highly cirrhotic milieu and limit anti-PD-1 therapy [164]. Importantly, these NETs can also directly play a role in immune escape by inhibiting the cytotoxic activity of CD8^+^ T cells [165]. PRSS35, an extracellular protease, was found to prevent the production of NETs via the cleavage of CXCL2 on neutrophils to suppress HCC tumor growth [166]. Additionally, GSK3A was found to assist in the infiltration of neutrophils and the formation of NETs [167]. Xin et al. developed a predictive model in which certain NET biosignatures could be used as prognostic and/or predictive immunotherapeutic response factors [168].

CXCR2 is a chemokine receptor found (but not limited to) in neutrophils that is commonly understood to aid in the recruitment of TANs into the HCC-ME. As a CXCR2 ligand, CXCL1 was found to be upregulated within HCC cells in response to acetyl-CoA accumulation and consequently led to TAN recruitment, favoring tumor growth/metastasis [169]. CXCR2-mediated neutrophil recruitment can also be achieved by CXCL2 [170]. IL-8 (also known as CXCL8) is the most important ligand for CXCR2 and CXCR1 on neutrophils in humans, and interestingly, self-sustaining IL-8^+^ neutrophils were identified recently in alcohol-associated hepatitis [171, 172]. It would be interesting to examine whether self-sustaining IL-8^+^ neutrophils also exist in HCC and play a critical role in HCC pathogenesis. It was also reported that a specific subset of ILC2s can recruit neutrophils to promote immunosuppression [173]. Compared with PD-1 monotherapy, combined immunotherapeutic targeting of CXCR2^+^ neutrophils (AZD5069, a CXCR2 inhibitor) and PD-1 in a MASH-HCC orthotopic mouse model resulted in increased survival [174]. Finally, CXCR2-mediated recruitment of TANs into the lung by SPP1 and the subsequent production of NETs were shown to be essential in premetastatic niche formation and HCC-derived lung metastasis in an orthotopic HCC metastasis mouse model [175].

Many studies have also demonstrated the relationship between neutrophils and anti-PD-1 therapies. For example, tumor cells can induce a specific subset of neutrophils (CD10^+^ ALPL^+^) that are associated with heavy CD8^+^ T-cell immunosuppression, which leads to resistance to anti-PD-1 therapy [176]. Interestingly, many patients exhibiting high resistance to PD-1 therapy have elevated levels of serum amyloid A (SAA), which induces the expression of PD-L1 on TANs via the STAT3 pathway [177]. TANs can be recruited through the upregulation of the CRKL/β-catenin pathway to further confer resistance to the same therapy [178]. However, a study using a subcutaneous HCC mouse model revealed that combined therapy with cabozantinib and anti-PD-1 therapy results in significantly better therapeutic efficacy, mainly due to increased neutrophil recruitment in parallel with decreased numbers of PD-1^+^ cells and T_regs_. This highlights the heterogeneous nature of neutrophils in the context of HCC [179]. A novel nanovaccine-based approach was also demonstrated to indirectly switch protumor neutrophils toward an antitumor phenotype [180].

#### Myeloid-derived suppressor cells (MDSCs)

MDSCs are a heterogeneous group of immature myeloid immune cells that have immunosuppressive functions in the context of many cancers and can be broadly categorized into monocytic (M)-MDSCs and polymorphonuclear/granulocytic (PMN)-MDSCs on the basis of their phenotypic and functional properties. They were first described as T-cell [181] and NK cell response suppressors as early as 2008 [182]; hence, their consequent association with poor prognosis. MDSCs can be characterized by several biomarkers, including but not limited to CD14^+^, CD11b^+^, CD15^+^, HLA-DR^-/low^, and CD33^+^ in humans and CD11b^+^, Ly6G^+^, Ly6C^+^, Gr-1^+^, and CD244^+^ in mice [183]. Furthermore, IL-6, CXCL1, CCL2, and CD40 are well-studied inducers and/or recruiters of MDSCs [184–188]. It is further imperative to note the similarities between MDSCs and TAMs and TANs, since they are all derived from myeloid progenitor cells (MPCs) and have overlapping immunosuppressive phenotypes, and TAMs/TANs can derive themselves from M-MDSCs/PNM-MDSCs [189–191].

MDSCs regulate HCC development by exerting their immunosuppressive function through many different mechanisms depending on their subset, since M-MDSCs harbor macrophage-like and PMN-MDSCs harbor neutrophil-like immunomodulatory functions in HCC. For example, PMN-MDSCs suppress T-cell proliferation/function by secreting arginase-1 (ARG1) to reduce the level of *L*-arginine and produce ROS, whereas M-MDSCs produce TGF-β1 to inhibit NK cell-mediated cytotoxicity [192]. The recruitment of MDSCs is aided by cancer stem cells (CSCs), notably through the LAPTM4B‒CXCL8 axis [193]. Furthermore, IL-37-treated orthotopic HCC murine models have significantly reduced immunosuppression due to the increase in aerobic glycolysis in MDSCs [194, 195].

Taken together, the use of various biomarkers, inducers and effectors of MDSCs as therapeutic targets via monoclonal antibodies/antagonists is a promising research field in the treatment of HCC. For example, CCL1^+^ MDSCs recruited by CCL15 have a wide range of immunosuppressive properties, highlighting the potential targeting of this chemokine [196]. Furthermore, Wen et al. demonstrated that the deletion of squalene epoxidase (SQLE) results in improved HCC outcomes through a decrease in MDSCs [197]. In addition, the YT521-B homology m^6^A RNA-binding protein-1/enhancer of zeste homolog-2 (YTHDF1/EZH2) axis has been shown to aid in the recruitment of MDSCs through the upregulation of IL-6, another axis to be considered in HCC immunotherapy [198, 199]. Preclinical trials with tivozanib, a multikinase inhibitor that indirectly decreases MDSC levels, have also shown great promise [200]. Since they also modulate the PD-1/PD-L1 pathway, combined therapeutics of MDSCs with ICIs also have great potential [201, 202].

#### Mucosal-associated invariant T (MAIT) cells

MAIT cells are a subset of innate-like T cells found in several mucosal sites and organs that display both innate and adaptive traits, mostly due to the presence of a semi-invariant T-cell receptor (TCR) that is coupled with a TCR β-chain that recognizes riboflavin metabolites (e.g., 6-formylpterin) that are not produced in homeostatic human metabolism [203]. These metabolites are presented on the surface of infected cells through the major histocompatibility complex class-I (MHC-1)-related protein-1 (MR1) receptor, allowing MAIT cells to produce an innate antimicrobial response. They have two modes of activation (1): MR1-dependent activation consists of TCR stimulation by MR1-presented antigens on infected cells to produce an inflammatory response toward bacterial/fungal infections, and (2) MR1-independent activation consists of the recognition of proinflammatory cytokines such as IL-12 and IL-15 secreted by infected cells to produce cytotoxic and inflammatory molecules against mainly viral infections [204]. The role of different subtypes of MAIT cells has been correlated with several types of cancer [205], including HCC [206, 207].

The literature has focused on the protective or malignant properties of MAIT cells in HCC because of their highly heterogeneous nature [208]. Some studies have noted decreased infiltration levels of MAIT cells in HCC patients [209, 210]. On the other hand, in addition to inactivating CD8^+^ T cells through the PD-1/PD-L1 axis, TAMs were also found to be involved in the suppression of MAIT function through this same cell‒cell interaction [99]. Furthermore, MAIT cells produce TNF to activate the immunosuppressive TNF receptor superfamily member-1B (TNFRSF1B) on T_regs_ to confer resistance in the context of combined lenvatinib + anti-PD-1 therapy [211]. MAIT cell-produced TNF-α can also activate quiescent HSCs into fibrotic myofibroblasts, which might favor the initiation of HCC [212]. In addition to activating the immunosuppressive phenotype of T_regs_, certain MAIT cell subtypes have immunosuppressive properties themselves [213]. Finally, MAIT cells are promising immunotherapeutic tools for the specific lysis of tumor cells, as successfully demonstrated by the engineering of HBV-targeting TCRs [214].

#### Natural killer (NK) cells

NK cells are group 1 ILC3s that play key roles in antiviral and antitumor immune responses through an effector cytotoxic phenotype. This cytotoxicity is not limited by the presentation of non-self-antigens through MHC-I on the surface of infected/cancer cells, which differs from that of cytotoxic CD8^+^ T cells. This cytotoxic effector function is mediated by the perforin-granzyme system, as well as the production of several inflammatory cytokines, such as IFN-γ and TNF-α, depending on the recognition of shifting activating/inhibiting signals on the surface of virally infected and tumor cells.

However, this normally highly efficient antitumor response might be circumvented by HCC-ME, which inhibits the infiltration of NK cells and induces a dysfunctional phenotype within the TME [215]. Some studies have linked the presence of CD49a-expressing NK cell subsets in intratumoral regions to low cytotoxicity [216, 217]. Moreover, TIM-3 and CD38 are gradually more highly expressed on the surface of NK cells in patients with HCV-induced cirrhosis who later develop HCC than in patients who do not develop HCC [218]. Importantly, TGF-β, and most likely the resulting fibrotic milieu, seems to interfere with the cytotoxic function of NK cells within HCC tumors [219]. Importantly, a recent study showed that, compared with littermates fed a normal diet, mice fed a high-cholesterol diet or ApoE knockout (which results in high cholesterol) had significantly lower tumor burdens after DEN injection or orthotopic HCC cell line implantation [220]. NK cell dysfunction and exhaustion are widely observed phenotypes within HCC tumors. Analysis of human transcriptomic data revealed that FBFBP2 is a marker of NK cell exhaustion in HCC tissues [221]. Additionally, NR4A1 was found to be involved in NK cell dysfunction in human HCC patients [222]. Moreover, the overexpression of TM4SF5 resulted in NK cell exhaustion and enhanced fibrotic hepatocarcinogenesis [223]. From a cell-to-cell perspective, it was further surprising that HSCs are involved in NK cell exhaustion [224].

TDEs are known to decrease NK cell activation and cytotoxicity in various cancer types [225]. One example of this phenomenon is tumor-derived exosomal circUHRF1, which drives exhaustion and decreases NK cell activity in HCC patients, whereby circUHR1 indirectly upregulates the expression of TIM-3, a molecule involved in T-cell exhaustion [226]. Single-cell resolution analysis revealed that NK cells lose their cytotoxicity as they approach the HCC tumor site more closely, as a result of a different cytokine gradient in the immunosuppressive milieu [227]. In addition, EpCAM^high^ stem-like cancer cells can evade NK cell-mediated immune surveillance via CEACAM1, a transmembrane glycoprotein that potentially acts directly on the inhibitory receptors of cytotoxic cells [228].

Generating new cutting-edge immunotherapies by using the natural cytotoxic phenotype of NK cells to our advantage and/or finding ways to overcome known pathways leading to their dysfunction is an interesting field of research. Known methods, such as CAR-NK cell therapy (similar to CAR-T-cell immunotherapies), are used alone or in combination with other forms of antitumor therapies [229, 230]; however, additional discoveries have been made using different targets and/or techniques. For example, combined sorafenib plus NK cell therapy was successful in an HCC rat model [231]. NK cell therapy was successful in preventing HCC recurrence in combination with a tumor acidity neutralizer and the lysis of NETs [232]. In addition, the inhibition of the Siglec-9 pathway within NK cells resulted in the amelioration of cytotoxicity [233]. It was also recently discovered that blocking CD47 resulted in the recruitment and activation of NK cells through DC-secreted IL-12 and CXCL9 [138]. Furthermore, the upregulation of RAC1 in infiltrated NK cells resulted in increased RAC1 activation, highlighting its potential use in immunotherapy [234]. The combination of sorafenib treatment with the transcriptional knockdown of STAT3 in a subcutaneous tumor model resulted in increased NK cell infiltration with enhanced antitumor activity [235]. Moreover, supplementation with the metabolic coenzyme nicotinamide adenine dinucleotide (NAD^+^) and its precursor nicotinamide mononucleotide (NMN) significantly increased NK cell cytotoxicity in an in vitro model [236]. Another recent study revealed that circulating tumor cells evade NK cell-mediated immune surveillance through the CD155-TIGIT axis, resulting in HCC metastasis [237]. A recent in vivo study further revealed that IL-15-mediated activation and infiltration of NK cells significantly increased the efficacy of combined anti-PD-1 and anti-TIGIT therapy, revealing the importance of normal NK cell activity in immunotherapy [238]. Another study demonstrated the positive effect of 2,5-dimethylcelecoxib (DMC) on the cytotoxic activity of NK cells within HCC tumors [239]. In addition, mice treated with a HIF inhibitor presented increased infiltration of NK cells in parallel with better outcomes in response to PD-1 therapy [240]. Finally, a very notable study revealed the efficacy of an NK cell engager molecule that directs the cytotoxic activity of NK cells to target glypican-3-expressing HCC cells [241].

#### Natural killer T (NKT) cells

NKT cells are a subset of innate-like T lymphocytes that are restricted to the recognition of CD1d on the surface of APCs and express both NK-like and T-like molecules that distinguish them from these two distinct cells. Under normal functional conditions, NKT cells secrete proinflammatory cytokines such as IFN-γ, IL-2, and TNF-α. Logically, these cells play a widely understood role in antitumor immunity, similar to NK cells and T cells, and the presence of exhausted NKT cells is similarly observed in the TME of several cancer types [242]. To underscore this notion, a recent study indeed revealed this altered NKT state within the microenvironment of both MASLD-HCC mouse models and human patients [243]. This might explain the correlation between the pattern of increased presence of NKT cells compared with that of NK cells and tumor progression reported in MASLD-HCC patients [244]. Furthermore, anti-PD-1 therapy in HCC patients results in the rescue of this exhausted phenotype [245]. Its IFN-γ-related antitumor apoptotic effect can also be enhanced by IRF-1 [246].

#### Innate lymphoid cells (ILCs)

ILCs are tissue-resident immune cells that can generally be divided into three subpopulations, namely, type-1 ILCs (ILC1s), ILC2s, and ILC3s, which are found mainly in mucosal tissues to aid in the innate immune response toward microbes and cancer. ILC1s are thought to functionally mirror Th1 cells, ILC2s mirror Th2 cells, and ILC3s mirror Th17 cells [65]. Importantly, NK cells are also broadly considered part of the ILC1s; accordingly, the mention of ILC1s in this section only relates to CD127^+^ ILC1s and not NK cells. Importantly, ILC1s constitute ~30% of all liver-resident lymphocytes, with ILC2s and ILC3s rarely being observed under homeostatic conditions, making ILC1s especially important potential modulators of HCC-ME [247]. ILC2s express neutrophil-attracting chemokines such as CXCL2 to induce immunosuppression in HCC [173]. Interestingly, the TME can influence the plasticity of NK cells switching into tumor-promoting ILC2s and/or ILC3s directly within the HCC tumor site [227]. B-cell-induced ILC2 differentiation has also been observed in human HCC [248]. A recent study revealed the ability of gut microbiome-derived metabolites to decrease IL-17A secretion by ILC3s, with the latter being a poor prognostic factor in patients with HCC [70]. In general, ILCs are not well studied in the context of HCC, as opposed to NK cells.

### Influence of adaptive immune cells on HCC development

#### CD4^+^ T cells

The hepatoprotective role of CD4^+^ T cells against HCC has been extensively studied [249]. Epigenetic changes can further drive this immune surveillance potentiation by enhancing the activation of CD8^+^ T cells [250]. Although the T_H_1/T_H_2 model has tended to be outdated over the past few years, CD4^+^ T cells are still commonly classified according to this model [251], with both effector phenotypes studied for their respective roles in the HCC-ME. T_H_1 cells are involved in microbial defense by potentiating macrophage phagocytosis and the proliferation of CD8^+^ T cells, notably through the secretion of cytokines such as IFN-γ, whereas T_H_2 cells are involved in an antiparasitic humoral adaptive immune response by inducing the proliferation of B cells while secreting cytokines such as IL-4 and IL-13. Other notable members of this group that are correlated with the onset and progression of HCC include T_H_17 cells and T_regs_ (the latter is discussed in more detail later). T_H_17 cells are CD4^+^ IL-17^+^ T cells differentiated from naïve CD4^+^ T_H_0 cells through the action of cytokines such as IL-6, TGF-β1, IL-21, and IL-1β, which in turn activate downstream transcription factors such as STAT3, RAR-related orphan receptor γt (RORγt) and RORα, which are all essential for the expression of T_H_17-specific cytokines and can potentially be considered treatment options in the context of HCC [252]. In addition to being involved in HCC, Th17 cells and their cytokines are involved in many inflammation-induced diseases, such as rheumatoid arthritis (RA), psoriasis, inflammatory bowel disease (IBD), and other autoimmune diseases. The presence of Th17 cells is commonly associated with poor prognosis and overall survival (OS) [244] and is implicated in all major steps of tumorigenesis.

The IL-17 family is a proinflammatory cytokine family, with IL-17A being the most abundant member that signals through its ubiquitously expressed heterodimeric receptor IL-17RA/IL-17RC and is produced mainly by Th17 cells but also includes γδ T cells, Tc17 cells (CD8+IL-17^+^ T cells), NKT cells, MAIT cells, and ILC3s. Under homeostatic conditions, the functions of IL-17 include antimicrobial peptide production, neutrophil recruitment and maintenance of barrier surface tissue integrity [253]. The role of IL-17 and T_H_17 cells in the immunopathophysiology of HCC has been well established in recent decades [254]. The association of IL-17 with known proinflammatory and tumorigenic signaling pathways, such as the NF-κB, mitogen-associated protein kinase (MAPK) and STAT3 pathways, is strongly correlated with various hallmarks of cancer, such as neoangiogenesis, inflammatory cytokine production and immunosuppression [255], and its scarcity or absence is widely shown to be a positive prognostic marker in patients receiving or not receiving treatment [256]. IL-22 is a mostly proinflammatory cytokine that is produced by T_H_17 cells, T_H_1 cells, T_H_22 cells, γδ T cells, NKT cells and ILC3s and signals through its heterodimeric receptor IL-22R1/IL-10R2 on hepatocytes and downstream pathways, such as the STAT3, MAPK and p38 pathways. Its endogenous hepatic function is involved in the tissue regenerative properties of the liver and antimicrobial defense; it has a dichotomous effect in the context of HCC [257] but is commonly associated with a poor prognosis [258, 259]. Although the tumorigenic effects of IL-22 on HCC are less established than those of IL-17, some studies have been able to link its proliferative properties and its protumor downstream pathways to HCC.

IL-17 is known to increase the expression of profibrotic genes such as TGF-β1, indicating its potential contribution to the induction of cirrhosis and, consequently, HCC. Circulatory IL-17 levels are notably useful in predicting HCC in its earlier stages in human patients with cirrhosis. For example, the deletion of IL-17 signaling results in the attenuation of fibrosis by decreasing inflammatory cytokine and STAT3-mediated collagen production, whereas the deletion of IL-22 signaling results in the exacerbation of fibrosis [260]. IL-17A has also been shown to act on HSCs directly by inducing collagen production and indirectly by mediating the release of HSC-activating cytokines such as TGF-β1 [260]. Inhibiting the IL-17 pathway also alleviates MASH symptoms and prevents the induction of HCC in mice [261, 262]. This preventative outcome was also replicated in ASH through an extensive study performed by Ma et al., who reported that steatotic IL-17RA-deficient hepatocytes result in decreased steatosis and fibrosis through defective TNF-TNFR1-dependent cholesterol synthesis [263, 264]. Moreover, the pharmacological inhibition of rho-associated kinase-2 (ROCK2) resulted in a significant decrease in induced fibrosis in mice, in parallel with reduced levels of phosphorylated STAT3 (p-STAT3), RORγt and IL-17A [265]. To counteract the STAT3-induced tumorigenic properties of IL-22, neutrophil-produced IL-22 binding protein (IL-22BP) functions as a soluble receptor that inhibits the binding of IL-22 to its transmembrane receptor, highlighting the potential therapeutic use of IL-22BP in HCC [266]. Finally, previous genome-wide association studies (GWASs) further associated IL-17 with an increased risk of MASLD [267]. Interestingly, IL-17RA^-/-^ murine models also tend to suppress excessive alcohol consumption through increased IL-17A levels in the brain [268], which potentially correlates with the tumor-promoting role of IL-17 even earlier than the acute hepatitis or cirrhosis stages and thus provides more insight into the behavior changes related to IL-17. Furthermore, IL-23-stimulated NCR^-^ ILC3s produce IL-17 in vivo, as do other types of IL-17-producing cells [269]. Moreover, the inhibition of miR-383 (an upstream regulator of IL-17) significantly attenuated tumor cell growth in vitro [270]. Although IL-22 can be used as a potential prognostic factor in the context of HCC, its dose-dependent differing effects in all stages of hepatocarcinogenesis suggest that, compared with IL-17, IL-22 is at most a minor actor [257].

Cancer stem cells (CSCs) play a pivotal role in driving the malignancy of liver cancer in all stages of HCC development because of their stem cell-like properties [271]. Single-cell analysis has revealed biodiversity among different CSC subpopulations [272]. Tumor cell biodiversity may drive microenvironmental reprogramming in liver cancer [273, 274]. Ni et al. and Gasmi et al. reported that IL-17 is involved in the programming of hepatic progenitor cells (HPCs) into CSCs through indirectly inducing fibroblast activation protein (FAP) on HSCs [275] and through directly downregulating miR-122 on HPCs [276, 277]. In addition, CSC-induced IL-17A expression from lymphatic endothelial cells promotes the self-renewal of liver CSCs, resulting in a hepatocarcinogenic positive amplification loop [278]. IL-17 was also confirmed to stimulate CD47^+^ CSC proliferation [279], and a choline-deficient methionine-supplemented diet induced LPC expansion [280]. IL-22, on the other hand, is a known stimulator of STAT3-mediated LPC proliferation; however, its overexpression results in a decrease in fibrosis, again highlighting its dichotomous effect in the context of HCC [281].

The metastatic and angiogenic properties of IL-17 have been studied in various experimental models, such as through the discovery that CAF-secreted CXCL11 upregulates the expression of circUBAP2 within HCC cells, which counteracts the inhibitory effect of miR-4756 on IFIT1 and IFIT3 [125]. These latter molecules can then increase the secretion of IL-17 by HCC cells and consequently increase the migration of HCC cells [125]. Moreover, TDEs enriched with miR-4508 are involved in the formation of lung premetastatic niches to induce HCC-derived lung metastasis [282]. Furthermore, in vivo adoptive transfer of T_H_17 cells resulted in increased migration, proliferation and angiogenesis, as well as the formation of premetastatic niches [283]. IL-17 was also found to be correlated with EMT markers in the context of intrahepatic and pulmonary metastasis [284]. IL-17 has been characterized by multiple studies with different hallmarks of immune surveillance escape and immunosuppression. A specific subset of IL-17^+^ Tc17 cells lacking IFNγ expression has recently been correlated with T_reg_ infiltration, an indicator of immunosuppression and poor prognosis [285]. In this context, IL-17 can reverse the proapoptotic properties of IFN-γ and subsequently lead to the activation of the protumoral NF-κB pathway [286].

#### CD8^+^ T cells

CD8^+^ T cells, or CTLs, are among the most effective and specialized actors in the adaptive antitumor response, mainly because of their cytotoxic activity toward virally infected cells and tumor cells. They induce apoptosis in infected cells via the perforin-granzyme mechanism, as well as the Fas/FasL pathway. To counteract this cytotoxic effector function, HCC-ME has developed various mechanisms to induce exhaustion and dysfunction phenotypes in CTLs, as discussed in the previous sections. Many studies have characterized several molecules or mechanisms that contribute directly or indirectly to CTL dysfunction [287], such as MMP9 [288], PVRL1 [289], and circCCAR1 [290]. Furthermore, cholesterol sulfate originating from tumor cells can lead to T-cell exhaustion [291]. Another metabolite, itaconate, can also cause exhaustion by upregulating immune checkpoints [292]. Additionally, metabolites derived from methionine within cancer cells are correlated with T-cell exhaustion [293]. Since PD-1 is widely expressed in exhausted CD8^+^ T cells, anti-PD-1 therapy is a clinically effective strategy for certain patients [294]. Recently, the presence of CD103^+^ CTLs was correlated with poor HCC prognosis [295]. CTL infiltration can be enhanced by exosomal ADCY7-mediated *CCL5* upregulation within the TME [296]. Finally, the effectiveness of metformin in improving the CTL response was assessed [297].

#### Regulatory T cells (T_regs_)

T_regs_ are a subset of CD4^+^ T cells that maintain immune tolerance under homeostatic conditions and prevent autoimmunity. As their name suggests, they have regulatory effects on various types of innate and adaptive immunity, such as DCs and T cells. Since the liver is constantly subject to antigens originating from the portal vein and thus is highly prone to constant inflammatory activity, the presence of T_regs_ is an important mainstay for limiting the deleterious effects of excessive inflammation. They are phenotypically distinguished from other CD4^+^ T cells by their expression of FOXP3 and CD25, both of which are vital for their immunosuppressive effector function, as well as their expression of cytokines such as IL-10 and TGF-β. Taken together, the presence of T_regs_ within the HCC-ME is correlated with poor prognosis and reduced overall survival, whereas the T_reg_-to-T_eff_ (T effector cell) ratio is commonly used as a prognostic marker. A highly immunosuppressive subset of T_regs_ expressing CCR4 is highly engaged in HBV-induced HCC [298].

SOX12 can induce the recruitment of T_regs_ through the upregulation of CCL20 [299]. T_regs_ were also found to be differentiated from naïve CD4^+^ T cells by NETs in a MASH-HCC mouse model [162]. Lipid droplets can also aid in the CCL20-mediated recruitment of T_regs_ in HCC [96]. Moreover, GDF15 indirectly suppresses T_reg_-mediated immunosuppression by eliminating FOXP3 [300]. One of many ways in which lenvatinib works is by indirectly suppressing STAT5 phosphorylation to effectively decrease IL-2-mediated T_reg_ differentiation in the context of anti-PD-1 therapy [301].

#### B cells

B cells are lymphocytes that are activated through BCR-mediated antigen recognition and costimulatory signal reception to become antibody-producing plasma cells, the central players of adaptive humoral immunity. Several types of B cells exist, including B-1 cells, B-2 cells, and regulatory B cells (B_regs_). The role of B cells in the pathogenesis of HCC is mixed. The absence of these cells has been shown to promote HCC initiation [302]; moreover, the presence of B cells is correlated with poor prognosis in some HCC patients [303]. The heterogeneous functions of the different types of B cells are the probable explanation behind this mixed literature.

Naïve B cells are correlated with good prognosis in HCC patients [304]. Petriv et al. reported that some subsets of B_regs_ express anti-inflammatory factors such as PD-L1 and IL-10 in mice with MASLD and HCC [305, 306]. Intestinal B cells are correlated with HCC development in the context of MASH, which is observed in choline-deficient high-fat diet-fed mice [72].

## Innovative experimental models for studying the pathogenesis of HCC and its associated immune response

### Cutting-edge experimental techniques to study the impact of the immune HCC microenvironment

In summary, the liver TME is a highly heterogeneous and complex environment that contains various immune cell types, each with distinct and pleiotropic properties in the context of HCC. In recent years, many scientific breakthroughs have been made in the field of cutting-edge technology discoveries to study the TME, especially those that are able to study multiple biomarkers simultaneously and are enhanced with the use of artificial intelligence (AI) [Table 1] [307, 308].

**Table 1:** Overview of current cutting-edge techniques in HCC immune research

Technique	Methodology	Applications	References
Multiplex immunofluorescence (mIF)	Consecutive IF staining of formalin-fixed paraffin-embedded tissue sections	• Extracellular or subcellular location of HCC markers • Biomarker changes in different etiology-derived HCC	[112, 308–315]
Flow cytometry (FC)	Fluorescent labeling of cell-surface antigens subjected to laser excitation	• Transmembrane receptor analysis • HCC immune cell sorting • Immunophenotyping • Identification of novel HCC immune subsets	[119, 299, 316, 317]
Single-cell RNA sequencing (scRNA-seq)	cDNA library is prepared from isolated single cells for high-throughput sequencing	• Immune profiling • Immunophenotyping • Identification of novel HCC immune subsets	[318–320]
Multiomics (spatial proteomics, transcriptomics, and metabolomics)	Integrates protein, gene expression, and metabolite profiling with spatial resolution.	• Spatial distribution of immune cells • Immune-evasive niche identification • Linkage of metabolic rewiring with HCC progression.	[321–327]
Hepatocyte-derived organoids	In vitro generation of three-dimensional liver-like structures from isolated hepatocytes	• Drug discovery • Immunotherapy assays • Disease development	[328–335]

Multiplex immunofluorescence (mIF) has steadily increased in popularity because of its usefulness in the characterization of specific cell types via histology. With the use of known markers of different immune cell types already used in standard immunohistochemistry (IHC) or immunofluorescence (IF), mIF differentiates itself by allowing the analysis of multiple markers on the same tissue sections with successive staining–stripping protocols, with up to 18–20 different markers being able to be visualized on a single section [309, 310]. The use of mIF has been successful in characterizing the TME in the context of various liver-associated diseases, including alcohol-associated hepatitis [311], necrotic liver formation [312], and liver aging [313]. Owing to the complex nature of the liver TME, various aspects of HCC research, such as identifying the factors conferring resistance to immunotherapy [108, 112, 314], posttherapy HCC recurrence [315], and metastasis [316], have been implemented via mIF.

Another major technique used in the characterization of the immune TME in HCC research is flow cytometry (FC), along with its modified and/or improved counterparts, such as cytometry by time of flight (CyTOF) and imaging mass spectrometry (iMS). FCs have been used for several decades to characterize the different transmembrane biomarkers of individual cells and, notably, to isolate these cells for further analysis or subset identification [120]. CyTOF, an improved version of FC, uses isotopes instead of fluorescent dyes to allow for improved labeling but consequently cannot be used for cell sorting [300, 317]. On the other hand, iMS allows the analysis of markers on tissues instead of a cell suspension, adding a spatial parameter [318].

Single-cell RNA sequencing (scRNA-seq) has paved the way for HCC research in recent years. Various techniques, such as microfluidics, can be used to isolate single cells and isolate the RNA to be reverse transcribed to cDNA, building an extensive library that can be sequenced with high-throughput next-generation sequencing (NGS) methods, allowing for an improved and personalized glance into the complex heterogeneous HCC TME. Notably, scRNA-seq can be used to identify different transcriptional immune cell subsets residing in peritumoral or intratumoral HCC tissues and thus assess their respective roles in the immunopathophysiology of the TME [319]. The characterization of the transcriptional landscape of single cells between HCC patients and healthy donors provides a clearer and more specific view of the role of individual cell types in the development of HCC [320] and can be used to analyze the effects of immunotherapy response or resistance at the individual cell level [321].

Spatially resolved omics technologies, including spatial proteomics, transcriptomics, and metabolomics, are transforming cancer research by enabling the localization of molecular information within the native tissue architecture. In HCC, these tools are increasingly used to dissect the spatial organization and functional states of immune cells, shedding light on the mechanisms underlying tumor progression and immune evasion. Spatial proteomics techniques such as imaging mass cytometry have enabled high-resolution profiling of the immune TME in HCC, as exemplified by a study that constructed a single-cell atlas from formalin-fixed, paraffin-embedded tissues of patients with MASH-HCC, HBV- or HCV-related HCC, and healthy donors [322]. Another application of imaging mass cytometry revealed how recurrence in HCC is governed by dynamic spatial alterations in cell composition and immune cell interactions across adjacent normal tissue, tumor margins, and intratumoral regions, highlighting a distinct immunosuppressive microenvironment in relapsed tumors [132]. By integrating spatial proteomics and transcriptomics, recent research revealed two stromal archetypes in HCC distinguished by ECM composition, immune infiltration, and metabolic programming [323]. By integrating spatial transcriptomics with scRNA-seq and mIF, researchers identified a tumor immune barrier composed of macrophages and CAFs at the tumor periphery, which restricts CTL infiltration and is correlated with resistance to anti-PD-1 therapy in HCC [112]. Spatial transcriptomic analysis of circulating tumor cells via scRNA-seq revealed transcriptional heterogeneity across vascular compartments in HCC [324]. Using spatial transcriptomics, researchers identified the regional expression of chemokines within HCC [325], whereas another study demonstrated that the proximity of CD8^+^ T cells to tumor cells in HCC correlates with improved patient survival [326]. In parallel, metabolomic profiling linked to the gut microbiota revealed that microbe-derived metabolites, particularly bile acids, may modulate immunotherapy responses in patients with HCC, with shifts in microbial composition and metabolite production serving as early predictive markers of treatment efficacy [327]. Additionally, untargeted metabolomics of patient-derived tissues and blood revealed metabolic reprogramming in HCC, particularly affecting TAMs and contributing to immune evasion [328].

Finally, hepatocyte-derived organoids are currently very promising research tools for studying the structural complexity of HCC [329]. Recently, Zen et al. developed an organoid system constructed on top of microfluidic chips that allows for the trial of immunotherapy treatments [330]. In addition to immunotherapy, gene therapy is another example of an example of an HCC treatment to be tested on organoid-based systems [331]. However, another study used organoids to characterize lenvatinib resistance [332]. Lim et al. further developed a patient-derived xenograft organoid model containing endothelial cells to study their impact on the TME [333]. Organoids have also been used to test the apoptotic activity of desloratadine [334] and donafenib [335]. Furthermore, organoids can also be used to study the effects of certain mutations in triggering the initiation of HCC [336].

**Fig. 3:**
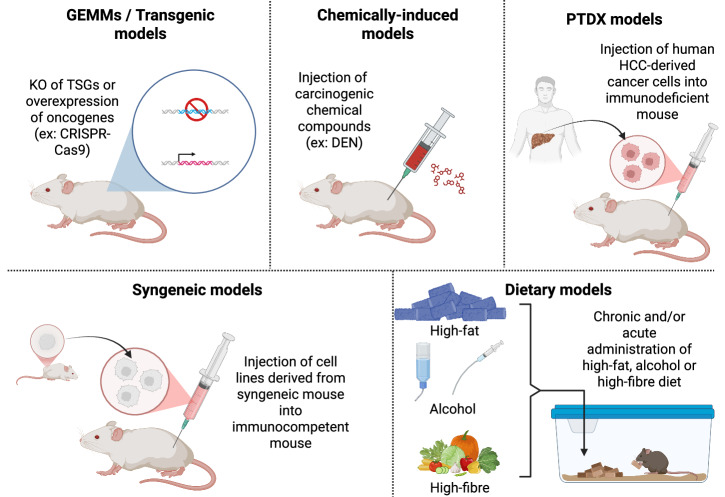
Experimental animal models used to study the impact of the immune-related HCC microenvironment. *Genetically engineered/transgenic mice:* Created via Cre-LoxP or CRISPR-Cas9 to overexpress or knock out oncogenes in hepatocytes, enabling studies of gene function and tumor-immune interactions. *Chemically induced models:* Tumors are initiated via the use of genotoxic (e.g., DEN) or nongenotoxic agents that mimic chronic liver injury, inflammation, and fibrosis. *Patient-derived xenograft (PDX) mice:* Human HCC tissues are implanted into immunodeficient mice, preserving tumor heterogeneity. *Syngeneic mice:* Mouse HCC cells are transplanted into genetically identical mice, allowing immune-competent tumor studies. *Dietary models:* Diets such as Western, alcohol, or high-fiber diets induce liver conditions that drive or modulate HCC development

Understanding the underlying mechanisms by which tumors escape immune surveillance is essential for improving current anticancer therapeutic strategies and patient outcomes. To this end, several experimental animal models mimicking clinical observations in patients have provided comprehensive information on how the immune microenvironment influences HCC initiation and progression [Table 2]. Several experimental models have been established that are particularly useful for studying the immune response and are suitable for validating, for instance, the efficacy of novel immunotherapeutic strategies [Fig. 3].

**Table 2:** Overview of experimental animal models in HCC immune research

Model	Principle	Advantages	Disadvantages	References
Experimental mouse models				
Genetically engineered mouse models (GEMMs)/transgenic models	Use of knock-in/knock-out models for GEMMs, and use of mutated oncogenes under liver-specific promoter for transgenic models.	• Immunocompetence. • Native TME.	• Limited mutational heterogeneity. • Long latency. • High-cost of breeding and caging	[177, 336, 337]
Chemically induced mouse models	Exposure to hepatic carcinogens to induce chronic liver damage and subsequent HCC.	• Immunocompetence. • Ease and cost-effective. • Tumor heterogeneity.	• Interindividual variability. • Long latency.	[338, 339]
Patient-derived xenograft (PTDX) mouse models	Ectopic or orthotopic transplantation of patient-derived HCC cells into immunodeficient mice.	• High clinical relevance. • Fast induction. • Preclinical drug testing.	• Immunodeficiency. • Engraftment rejection. • High-cost and technical difficulty. • Limited native TME.	[328, 333, 340]
Syngeneic mouse models	Ectopic or orthotopic implantation of murine HCC cell lines into immunocompetent mice.	• Immunocompetence. • Reproducibility. • Fast induction. • Cost-effective.	• Limited mutational heterogeneity. • Technical difficulty.	[341–343]
Dietary models				
Western-diet/high-fat diet	Chronic exposure to WD or HFD-diet to induce and/or study HCC in the context of fatty liver.	• MASLD/MASH modeling • Mimics natural HCC progression.	• Long latency. • Variable tumor incidence.	[162, 344–347]
Alcohol diet	Chronic and/or acute alcohol consumption to induce and/or study HCC in the context of ALD.	• ALD/ASH modeling. • Chronic inflammation and fibrosis.	• Long latency. • Variable tumor incidence. • High mortality risk.	[348–356]
High-fiber diet	Chronic exposure to high-fiber diet to study its impact on HCC.	• Study of the gut-liver axis in the context of HCC.	• Mild tumorigenic effect.	[357–361]

#### Genetically engineered/transgenic murine models of HCC

Genetically engineered mouse models (GEMMs) have been pivotal in mimicking the genetic and histological characteristics of human HCC, wherein oncogene or tumor-suppressor genes are activated or suppressed, respectively, to induce tumorigenesis within the native and immunocompetent hepatic microenvironments of various mouse strains. In addition to mating-related logistical shortcomings, the major disadvantage of this model is the resulting homogeneity of the tumor, since the number of genetically engineered genes within one mouse strain is limited and does not entirely reflect the heterogeneous nature of human cancer. Models such as the Alb-Cre mice used with LSL-TGFB1R and Myc-driven models allow for the investigation of specific mutations and their effects on immune cell infiltration and function within the TME [178, 337]. Transgenic models that express specific immune-modulating genes provide insights into the role of immune checkpoints and cytokine signaling within the TME. For example, MDR2-deficient mice present an altered tumor immune landscape, highlighting the role of immunosuppressive cytokines in HCC [338].

#### Chemically induced murine models of HCC

Chemical carcinogens, such as the genotoxic compound DEN, are commonly used to induce de novo HCC in mice and provide insights into the role of the immune system in hepatocarcinogenesis. It is commonly used in addition to CCl_4_ to effectively reproduce the cirrhotic HCC seen in human patients, thus allowing an improved analysis of immune HCC-ME interactions in a fibrotic milieu. For example, the DEN+CCl_4_ method was used here to analyze the impact of miR-223-induced decreases in PD-1 and PD-L1 expression on immune cell expression during hepatocarcinogenesis and tumor progression [339]. It is further plausible to study cancer stemness and immune evasion [340]. These models have demonstrated that inflammation and immune responses heavily influence tumor development and progression.

#### Patient-derived tumor xenografts (PTDXs)

PDTX models, in which human HCC tissues are implanted into immunocompromised mice, enable the study of the human immune microenvironment and the tumor response to immunotherapies. This model has shown promise in evaluating the role of immune checkpoints and responses to therapies [329]. Furthermore, the inhibition of specific genes in combination with PDTX in mouse models is an effective tool for studying HCC [334, 341]. However, the ability of these strains to fully recapitulate the TME is still very limited, since these mice are immunodeficient enough to avoid xenograft rejection.

#### Syngeneic mouse models

Although ectopic/subcutaneous tumor injection models are widely used because of their simplicity and reproducibility, they are limited in replicating HCC-ME due to their metastatic nature. Orthotopic engraftment of syngeneic cell lines into the hepatic subcapsular space is a much more reliable method to mimic and analyze HCC-ME since it promotes an intact immune system and allows the study of immune responses to tumors. Since the injected HCC cells are syngeneic with the host murine liver, the use of immunocompromised mice, as opposed to PTDX models, is not needed. They are particularly useful for evaluating the efficacy of immunotherapies such as PD-1/PD-L1 inhibitors [342, 343]. This method is also very advantageous because of the ability to implant HCC cells into mice of any strain harboring genetic knockouts or knock-ins of various immune cell types/molecules [344]. Furthermore, before being injected, tumor cells can also be genetically or extragenetically modified (with the use of plasmids or vectors) beforehand to study the impact of receptors/mediators expressed by HCC cells that impact the surrounding TME.

As multiple environmental factors are involved in and contribute to the development of chronic liver diseases associated with HCC development, several experimental animal models have been proposed to mimic the clinical observations made in patients [Fig. 3].

#### Western diets (high-fat-cholesterol-sugars)

The prevalence of MASLD and its advanced form, MASH, is drastically increasing in proportion with the rate of obesity in western and developing countries [9] due to the increase in food processing and shelf stabilization of foods. Both etiologies present distinct immunologic profiles that can then lead to hepatocarcinogenesis and tumor progression; hence, characterizing the immune changes induced by chronic consumption of a Western diet is imperative. Notably, mice fed a Western diet presented increased levels of infiltrating lymphocytes within the liver and a significant increase in HCC growth [345]. Furthermore, mice fed a choline-deficient high-fat diet and induced HCC with DEN, in parallel with Western diet-fed mice, harbored increased amounts of Tregs and infiltrated neutrophils prior to the development of HCC, resulting in an ideal carcinogenic niche [162]. Moreover, mice fed a Western diet + CCl_4_ injection lacking HILPDA expression in hepatocytes had delayed HCC growth and fewer exhausted T cells than did mice fed a control diet [346]. Some MASH-HCC models were developed by feeding mice a high-fat-fructose-cholesterol diet and subsequently injecting DEN, and drastic increases in TAM and CD8^+^ T-cell dysfunction can be observed within the TME [347]. The consumption of a high-fat diet notably modulates known oncogenic pathways, such as those associated with the palmitoylation of AKT, an extensively studied hepatic tumor inducer [348]. Most notably, Zhang et al. discovered that a high-fat high-cholesterol diet, not a high-fat low-cholesterol diet, drives the formation of MASLD-HCC through gut dysbiosis [68].

#### Alcohol

The correlation between alcohol consumption and the incidence of HCC has been characterized in many studies and meta-analyses for several years [349, 350]. Like MASH-induced HCC, the prevalence of alcohol-induced HCC has been steadily increasing with the development of low-income countries and the gradual reduction in viral hepatitis incidence due to antiviral therapies/vaccination efforts [10]. Ethanol and its metabolites have immunomodulatory effects in the hepatic microenvironment to favor HCC. For example, alcohol induces gut permeability, resulting in increased translocation of bacterial endotoxins to the liver, in turn triggering an innate immune response that can potentially play a role in the development and/or progression of HCC [351, 352]. KCs are the primary immune cells that trigger inflammation by releasing proinflammatory cytokines and chemokines, which leads to the infiltration of circulatory immune cells such as monocytes, neutrophils, and T cells. The same KCs also generate ROS to further damage hepatocytes and lead to lipid peroxidation-induced mutagenesis [353, 354]. Furthermore, chronic alcohol consumption favors the metabolism of ethanol into acetaldehyde by CYP2E1 instead of alcohol dehydrogenase, a pathway that also generates ROS as a metabolic byproduct [355]. In the precancerous liver, acetaldehyde might also play a role in the development of fibrosis through HSC-induced ECM deposition [356]. Furthermore, the formation of DNA and protein adducts by acetaldehyde [357] can also, in theory, affect immune cells in parallel with hepatocytes to cause their transformation. Recently, alcohol has been found to exacerbate the carcinogenic properties of the cyclic GMP-AMP synthase (cGAS)-stimulator of interferon genes (STING) pathway by stimulating the transcription of proinflammatory interferons and interleukins [141].

#### High-fiber

Since dietary fiber cannot be digested and absorbed by the digestive system, it is an important nutritious source for the gut microbiome; specifically, fermented low-sugar fiber-containing foods are overwhelmingly considered a healthy carbohydrate choice. Although the literature suggests that fiber generally has a hepatoprotective effect [358, 359], the gut microbiome is indirectly connected to the liver through the portal vein, with gut-derived secondary metabolites and antigens potentially affecting the initiation and growth of HCC. Accordingly, the consumption of the fermentable fiber inulin in dysbiotic mice resulted in hepatocarcinogenesis [360]. This effect was later shown to be preventable by the use of vancomycin through the modulation of the gut microbiota and its metabolism [361]. Another study further showed that an inulin-rich diet in cholemic mice ultimately resulted in hepatic tumor growth [362].

## Emerging therapeutic strategies for HCC treatment

### Conventional HCC treatments

**Fig. 4:**
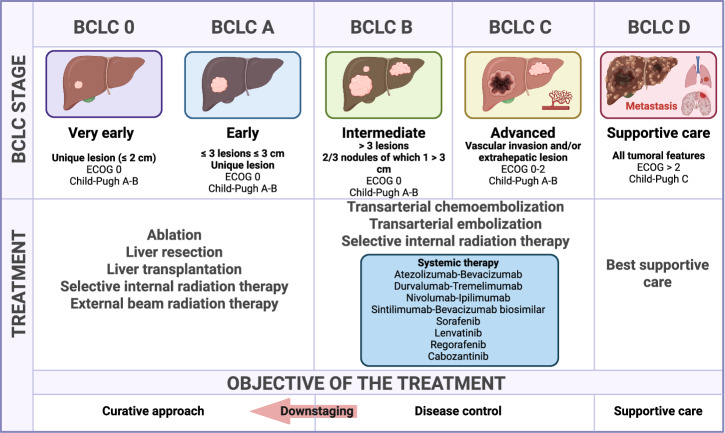
Barcelona Clinic Liver Cancer (BCLC) staging system and corresponding treatment strategies for hepatocellular carcinoma (HCC). This figure illustrates treatment allocation according to tumor burden, liver function (Child‒Pugh classification), and performance status (ECOG: Eastern Cooperative Oncology Group). Curative approaches—including liver resection, ablation, and transplantation—are recommended for early-stage disease (BCLC 0–A). Intermediate-stage HCC (BCLC B) is typically managed with transarterial therapies; however, systemic therapy may also be considered a first-line option in selected patients with high tumor burdens or who are unsuitable for transarterial therapies. Advanced-stage disease (BCLC-C) is treated with systemic agents, including immunotherapy-based regimens such as atezolizumab–bevacizumab, durvalumab–tremelimumab, and nivolumab–ipilimumab. End-stage disease (BCLC D) is managed with best supportive care. Treatment goals vary by stage and include curative intent, disease control, downstaging, or symptom management. Notably, the integration of immune checkpoint inhibitors with locoregional therapies such as TACE is an emerging strategy under active investigation, particularly in intermediate-stage HCC, and may further refine future therapeutic algorithms

Many treatments for HCC are available, and the choice of their applicability depends on HCC features, underlying liver function and patient comorbidities [Fig. 4] [Table 3]. When HCC is localized within the liver, locoregional treatments such as liver resection (LR), ablation, TACE, selective internal radiation therapy (SIRT) or external beam radiation therapy are preferred. In contrast, in cases of vascular invasion or extrahepatic spread, systemic therapy is recommended as a first-line treatment. In the setting of liver failure, locoregional or systemic treatments are contraindicated due to their potential toxicity and high risk of complications; in this case, only liver transplantation (LT) is considered a curative treatment, but only in selected patients with HCC and a low risk of recurrence [363–366]. Choosing the most appropriate treatment for patients can be complicated, and algorithms such as the BCLC algorithm or international guidelines are available to assist clinicians in their choice. The development of new therapeutic approaches as well as the recent positive results of immunotherapy in HCC have allowed clear improvements in patient prognosis and the treatment of more fragile patients in terms of liver function, age and associated comorbidities.

**Table 3:** Results from positive Phase 3 clinical trials used in first-line therapy studiesres

Phase 3 clinical trials	IMBRAVE 150		HIMALAYA		Checkmate 9DW	
Treatment	Atezolizumab–Bevacizumab	Sorafenib	Durvalumab–Tremelimumab	Sorafenib	Nivolumab–Ipilimumab	Sorafenib
Overall survival (median) months	19.2	13.2	16.4	13.8	23.7	20.6
Overall survival rate at 24 months	-	-	40.5%	32.6%	19%	39%
Overall survival rate at 36 months	-	-	30.7%	19.8%	38%	24%
Overall survival rate at 48 months	-	-	25.2%	15.1%	-	-
Disease-free survival (median) months	6.8	4.3	3.8	4.1	9.1	9.2
Objective response rate	30%	11%	20%	5%	36%	13%

The recent positive results from the interim analysis of the Phase 3 IMbrave050 trial, in which patients at high risk of HCC recurrence following LR or ablation were randomized to receive atezolizumab plus bevacizumab for 12 months after curative treatment or surveillance (recurrence-free survival: 0.72, 95% CI 0.56–0.93), prompted a reconsideration of adjuvant therapies for this high-risk population [367]. However, the latest update presented at ESMO 2024 did not confirm the initial positive findings regarding recurrence-free survival rates, and adjuvant therapy is not recommended after LR [363]. However, another randomized phase 2 study conducted in China explored the efficacy of sintilimab as an adjuvant treatment in patients who underwent surgery for HCC with microvascular invasion identified in surgical specimens (a necessary criterion for inclusion). In this trial, 198 patients were randomized to receive either sintilimab (*n* = 99) or surveillance (*n* = 99). After a median follow-up of 23.3 months, the results revealed that sintilimab significantly prolonged recurrence-free survival compared with surveillance alone (median recurrence-free survival of 27.7 months versus 15.5 months; HR = 0.534, 95% CI: 0.360–0.792; *p* = 0.002) [368]. While these results are promising, additional data are needed to evaluate the impact of these strategies on OS and to determine whether adjuvant therapies can be integrated as a standard treatment option for patients with early-stage HCC at high risk of recurrence. Furthermore, neoadjuvant approaches combining systemic treatment followed by locoregional therapy are underway [369], with encouraging early-phase 2 results. Combinations with other HCC treatments, such as endoarterial treatments, can also be proposed to increase treatment efficacy, and ongoing trials are assessing the impact of adding systemic treatments such as tyrosine kinase inhibitors (TKIs) or ICIs to ablation.

TACE is the first-line treatment for intermediate-stage disease, which includes unresectable multinodular lesions without vascular invasion or extrahepatic spread in patients with preserved liver function. TACE can also be used in early-stage HCC when curative treatment is not feasible or has failed and as waiting or downstaging treatment in cases of LT [370]. A recent systematic review of cTACE efficacy that included a total of 10,108 HCC patients revealed that the median OS was 19 months and that the 5-year OS rate was 32% [371]. An emerging strategy for treating HCC involves combining systemic treatments with locoregional therapies to improve response rates in HCC patients. The combination of locoregional therapies and ICIs leverages localized tumor necrosis induced by locoregional therapies, which release tumor-associated antigens and promote immune activation, whereas ICIs enhance this response by blocking immune checkpoint pathways, thereby overcoming immune exhaustion and enabling a sustained antitumor immune response. Several trials have investigated combinations of TKIs ± ICIs with TACE, but these studies have not achieved their primary endpoints. The TACTICS study, conducted exclusively in Japan, was an open-label trial with time to progression (until HCC became untreatable by TACE) as the primary endpoint, but it did not demonstrate an improvement in overall survival [372]. The Emerald-01 study, however, showed promising results. After a median follow-up of 17.4 months, the combination of TACE with durvalumab-bevacizumab demonstrated superior progression-free survival (PFS) compared with TACE with placebo, with an HR of 0.77 (95% CI: 0.61–0.98) and a median PFS of 15 months versus 8.2 months [373]. The results from the LEAP-012 study, presented this year in 2024, also revealed that the combination of lenvatinib-pembrolizumab and TACE significantly improved PFS compared with TACE alone in patients with intermediate-stage HCC (HR = 0.66, 95% CI: 0.51–0.84), with a median PFS of 14.6 months versus 10 months [374]. These results are encouraging, but long-term OS data for both studies are still awaited. Notably, grade 3–5 adverse events were more common in the TACE + systemic treatment groups than in the control group (31% versus 13% for EMERALD-01 and 71% versus 21.5% for LEAP-012). Further phase III trials evaluating TACE are ongoing. These findings suggest that combined therapies may soon become the standard of care for intermediate HCC patients, potentially enabling more patients to pursue curative options such as LR or LT.

#### Immune checkpoint inhibitors (ICIs)

The major change in HCC management in the last few years has been the recent positive results of immunotherapy. For a long time, TKIs were the only therapeutic option for advanced HCC. However, recent advances in clinical studies involving ICIs have profoundly transformed the management of this cancer. These treatments include anti-PD-1 agents such as pembrolizumab and nivolumab; anti-PD-L1 agents such as durvalumab and atezolizumab; and anti-CTLA-4 agents such as tremelimumab and ipilimumab. The IMbrave150 trial, a phase III study, compared the combination of atezolizumab and bevacizumab with sorafenib. The results revealed that atezolizumab and bevacizumab significantly improved OS (HR 0.58, 95% CI 0.42–0.79; *p* < 0.001) and PFS (HR 0.59, 95% CI 0.47–0.76; *p* < 0.001) [375, 376]. Similarly, the HIMALAYA trial evaluated the STRIDE regimen, which combines tremelimumab and durvalumab, against sorafenib. The primary endpoint of OS was achieved, with STRIDE demonstrating superior OS (median OS 16.43 months vs. 13.77 months; HR 0.78, 95% CI 0.65–0.93; *p* = 0.0035) [377]. A separate phase 2–3 study conducted in China assessed sintilimab (a PD-1 inhibitor) combined with a bevacizumab biosimilar (IBI305) versus sorafenib. This trial met its primary endpoint, showing that the combination provided better OS than sorafenib did (HR 0.57, 95% CI 0.43–0.75; *p* < 0.0001) [368]. More recently, the CheckMate 9DW trial (NCT04039607) reported achieving its primary endpoint of improved OS during an interim analysis. This trial evaluated the combination of ipilimumab and nivolumab with lenvatinib or sorafenib, further reinforcing the role of ICI-based therapies for advanced HCC [378]. Ongoing trials on the benefits of combining locoregional therapy with systemic therapy in selected patients will probably expand future indications for systemic therapy. Atezolizumab-bevacizumab was also associated with a complete response, which allows the consideration of LR and/or LT after downstaging in patients with initial advanced HCC not accessible to any curative treatment [379]. However, a major current question is determining the best first-line systemic treatment option between ICIs and TKIs. To date, no robust comparative studies are available to resolve the question of atezolizumab-bevacizumab versus durvalumab-tremelimumab or ipilimumab-nivolumab, as existing phase III trials have consistently used TKIs as control arms.

ICIs are generally well tolerated but can be associated with immune-mediated adverse effects, with an increased risk when two ICIs are used simultaneously. Owing to this risk, ICIs are not recommended for patients with moderate to severe autoimmune diseases, making TKIs the first-line treatment of choice for these patients [380]. Similarly, TKIs remain the preferred first-line treatment for liver transplant recipients experiencing recurrent HCC that requires systemic therapy. The mechanism of action of ICIs, which enhances T-cell activation, can lead to graft rejection. Indeed, liver graft rejection was observed in 29% of 52 transplanted patients treated with ICIs for various cancers, with a mortality rate of 13% [381].

The potential impact of liver disease etiology on the ICI response was first suggested in subgroup analyses from the IMbrave150 trial [375]. In this subgroup of patients treated with atezolizumab+bevacizumab, the HR was 0.91 (95% CI 0.52–1.60) for those with nonviral liver disease, whereas the HRs were 0.51 and 0.43 for the hepatitis B and C cohorts, respectively, suggesting poorer survival in the nonviral group. However, a recent meta-analysis of six randomized controlled trials using ICIs demonstrated that OS was significantly better in patients treated with ICIs than in those treated with TKIs, regardless of viral or nonviral etiology (hepatitis B: HR = 0.70, *p* < 0.001; hepatitis C: HR = 0.78, *p* = 0.04; nonviral: HR = 0.87, *p* = 0.02) [382]. Furthermore, a real-world cohort study revealed no significant difference in OS or PFS based on liver disease etiology [383]. Additional studies are needed, but current data do not preclude the use of ICIs in patients with MASLD.

The use of bevacizumab in systemic treatments for advanced HCC has raised concerns due to the risk of bleeding, particularly in patients with portal hypertension (PHT) and esophageal varices (EVs), given its anti-VEGF mechanism of action. In the IMbrave150 study, the risk of gastrointestinal bleeding was greater with atezolizumab+bevacizumab (2.4% for PHT-related bleeding vs. 0.6% with sorafenib) [375]. Importantly, patients included in the trial were required to have no high-risk EV and to undergo PHT-adapted prophylaxis. Real-world data report rates of PHT-related bleeding as high as 14%, especially in patients with a recent history of PHT-related bleeding or portal vascular invasion [384, 385]. The screening and management of PHT in patients with HCC are thus critical for reducing this risk. Upper endoscopy, such as nonselective beta-blockers (NSBB) when indicated, is recommended for all patients to identify EVs and implement prophylaxis [386]. Band ligation of EVs should be reserved for secondary prophylaxis or when NSBBs are contraindicated, as it may delay treatment initiation and increase the risk of bleeding secondary to the ligation procedure. However, the presence of large EVs or curative anticoagulation is not associated with an increased risk of bleeding under atezolizumab-bevacizumab, and this combination can be used in these cases with appropriate NSBB prophylaxis [384, 385, 387]. Thus, for patients at high risk of bleeding (with a recent history of PHT-related bleeding or extensive portal vein invasion), the combination of durvalumab-tremelimumab or TKIs could be a viable alternative. Nevertheless, it should be noted that data on the use of these options, especially durvalumab-tremelimumab, in patients with extensive vascular invasion remain limited since such patients were not included in the phase 3 study [377].

### Molecular approaches to predict immunotherapy efficacy in HCC patients

Molecular biomarkers offer insights into the molecular landscape of HCC, and their identification and targeting represent key steps to improve HCC patient outcomes. Currently, only 30% of patients with advanced HCC respond to ICIs, highlighting the need for predictive biomarkers to guide treatment decisions.

#### Tumor mutational burden (TMB)

A high TMB is correlated with improved immune responses in certain cancers. However, in HCC, high TMB is rare (0.8%), and no clear association with ICI response has been demonstrated [388].

#### Microsatellite instability (MSI)

MSI/dMMR tumors are linked to increased lymphocytic infiltration and improved ICI responses in various cancers. However, the MSI incidence in HCC is very low (<3%), limiting its applicability [388].

#### TP53 gene mutations

Mutations in the TP53 gene are associated with specific IFN-γ gene signatures, increased infiltration of Treg Foxp3+ cells, reduced CD8+ T-cell infiltration in HCC, and increased mortality rates among 369 HCC patients. These mutations could predict a poor response to immunotherapy [388].

#### *Wnt/β-*catenin signaling

In 40–80% of HCCs, dysregulation of Wnt/β-catenin signaling is associated with ICI resistance, shorter overall survival (9.1 vs. 15.2 months), and shorter progression-free survival (2.0 vs. 7.4 months) than in patients with nonmutated Wnt/β-catenin tumors. Wnt/β-catenin pathway genes may serve as predictive biomarkers for immunotherapy efficacy [389, 390]. In addition to tumor biopsies, PET scanning using choline is a promising approach for detecting Wnt/β-catenin-mutated tumors [391].

#### IFNAP signature

This 11-gene signature related to interferon‒gamma signaling and chemotaxis is correlated with anti-PD1 responses [392].

#### Circulating tumor DNA (ctDNA)

ctDNA consists of short DNA fragments released by tumor cells, providing insights into tumor mutational landscapes and acting as a liquid biopsy. In HCC, frequent somatic mutations affect pathways such as TERT (60%), TP53 (30%), CTNNB1 (20%), oxidative stress, and Akt/mTOR. Pilot studies have confirmed that ctDNA detection in HCC patients is strongly correlated with plasma and tumor mutations (e.g., TERT, TP53, and CTNNB1). The mutation burden increases with BCLC stage. Among patients treated with atezolizumab-bevacizumab, persistent ctDNA mutations or the emergence of new mutations correlated with disease progression, suggesting that ctDNA is a promising tool for evaluating systemic treatment response and detecting tumor subclonal selection [393].

#### Artificial intelligence (AI)

An AI model, the ABRS-P, was developed to predict progression-free survival in HCC patients treated with atezolizumab-bevacizumab via histological slides. When validated across multiple cohorts, the model demonstrated a significant association between high ABRS-P scores and improved treatment response [394].

#### PD-L1 expression

While PD-L1 serves as a predictive biomarker in other cancers, its utility in HCC remains controversial. Data from studies such as CHECKMATE 040, KEYNOTE 224, IMbrave150, and HIMALAYA indicate similar responses to ICIs regardless of PD-L1 expression levels [388].

### Enhancing the immunotherapy response with multimodal combinations

Only ~30% of liver cancer patients currently respond to immunotherapy, underscoring the need for more effective treatment strategies. To address this, emerging approaches increasingly focus on rational combination therapies that target complementary mechanisms of resistance. Among these, triplet regimens and vaccines are generating increasing interest. The ongoing PRODIGE 81–FFCD 2101–TRIPLET-HCC trial is evaluating the combination of atezolizumab, bevacizumab, and ipilimumab, with results pending [395]. Novel combinations are also under investigation. For example, in a phase 2 single-arm trial, the bispecific antibody KN046 (anti–PD-L1/CTLA-4) combined with lenvatinib achieved an objective response rate (ORR) of 45.5% [396]. Similarly, adding the TIGIT inhibitor tiragolumab to atezolizumab-bevacizumab resulted in a 43% ORR, whereas the ORR was 11% with atezolizumab-bevacizumab alone [397].

### TIGIT as a therapeutic target in HCC

T-cell immunoreceptor with Ig and ITIM domains (TIGIT) has emerged as a promising immune checkpoint in the HCC-ME. As an inhibitory receptor expressed on T cells and NK cells, TIGIT plays a crucial role in immune evasion by dampening antitumor immune responses, thereby contributing to HCC progression. Notably, a high baseline presence of intratumoral macrophages and T_regs_ has been associated with improved clinical outcomes in patients treated with a combination of atezolizumab (PD-L1 inhibitor) and tiragolumab (TIGIT inhibitor) but not with atezolizumab monotherapy. Preclinical mouse tumor models provide additional mechanistic insights, demonstrating that TIGIT blockade with tiragolumab surrogate antibodies induces an inflammatory shift in TAMs, monocytes, and DCs through Fc gamma receptor (FcγR) engagement. This, in turn, drives the reprogramming of antitumor CD8^+^ T cells from an exhausted effector-like state into a more memory-like phenotype, enhancing long-term immune surveillance. These findings underscore a novel mechanism by which TIGIT inhibitors can remodel the immunosuppressive TME, suggesting that FcγR engagement is a critical consideration in the development of next-generation anti-TIGIT antibodies [398]. Studies have shown that TIGIT is upregulated in HCC, particularly at advanced disease stages, and is often associated with poor prognosis, reduced T-cell effector function, and increased immune exhaustion [399]. Blockade of TIGIT, either as monotherapy or in combination with PD-1/PD-L1 inhibitors, has demonstrated potential in restoring T-cell cytotoxicity and enhancing antitumor immunity in preclinical HCC models [398]. Given the complex immunosuppressive landscape of HCC, targeting TIGIT—especially in combination with other ICIs or myeloid-targeting therapies—represents a promising strategy to improve treatment outcomes, particularly in patients with immune-resistant disease. Further research is needed to optimize combination approaches, explore predictive biomarkers, and evaluate long-term clinical benefits.

### Vaccine therapy for HCC

Therapeutic vaccines for HCC aim to stimulate the immune system to recognize and eliminate cancer cells in patients with existing disease. These vaccines utilize various approaches to achieve this goal, each differing in their mechanisms and production methods. Among these, peptide vaccines have gained attention for their ability to use small protein fragments corresponding to tumor-associated antigens (TAAs), such as glypican-3 (GPC3). These peptides specifically activate T lymphocytes, enabling them to identify and destroy tumor cells with minimal off-target effects. For example, a phase I clinical trial involving GPC3-derived peptides demonstrated their capacity to elicit immune responses in HCC patients without causing severe adverse effects [400]. Multiple peptide vaccines, such as GV1001, have expanded the scope of therapeutic vaccination by targeting multiple antigens simultaneously. GV1001 incorporates HCC-associated antigens such as alpha-fetoprotein (AFP), human telomerase reverse transcriptase, and melanoma-associated gene-A1. Clinical findings indicate that GV1001 administration reduces regulatory T cells (CD4^+^CD25^+^Foxp3^+^), which are known to suppress antitumor immunity, although no vaccine-specific immune responses are observed [401]. This limitation reflects the broader need to enhance the potency of therapeutic vaccines, particularly in the immunosuppressive microenvironment characteristic of HCC. Recognizing these challenges, researchers are exploring combinatory strategies to increase the effectiveness of peptide-based vaccines. One promising avenue is the integration of these vaccines with ICIs, which can counteract immune evasion mechanisms employed by tumors. For example, an ongoing phase II trial is evaluating the combination of a CD4 Th1-inducing telomerase-based cancer vaccine with atezolizumab and bevacizumab in patients with unresectable HCC [402]. This approach aims to synergize vaccine-induced immune activation with checkpoint blockade and antiangiogenic effects, offering a multifaceted strategy to improve therapeutic outcomes in HCC.

DC vaccines have also emerged as a promising strategy to enhance antitumor immunity in cancer patients. DCs are key APCs that process and present tumor antigens to T cells, promoting a targeted immune response. In the development of DC vaccines, dendritic cells are extracted, loaded with TAAs or tumor lysates ex vivo, and reintroduced into patients. These modified DCs travel to the lymph nodes, where they activate tumor-specific T cells, enhancing the immune response against the tumor. Clinical trials evaluating DC vaccines have shown encouraging results. For example, a phase I trial investigated a dendritic cell vaccine primed with AFP, a protein frequently overexpressed in HCC, using COMBIG-DC (ilixadencel). When administered intratumorally, the vaccine showed positive immunogenicity and was well tolerated, as evidenced by blood marker analysis [403].

Other innovative vaccine strategies are currently under development. DNA vaccines operate by delivering plasmid DNA encoding TAAs into host cells, thereby triggering the immune system to identify and eliminate cancer cells. Similarly, mRNA vaccines leverage messenger RNA to instruct host cells to produce tumor-associated proteins, which subsequently elicit an immune response. Early studies of mRNA vaccines targeting AFP or personalized neoantigens have demonstrated promising results, generating robust immune reactions. Tumor cell lysate vaccines represent another avenue, utilizing fragments of disintegrated tumor cells as a rich source of antigens to stimulate the immune system. When paired with DCs, these lysates significantly enhance antigen presentation and T-cell activation. Research has further revealed that lysates from HCC cells may prevent immune cell exhaustion, suggesting a novel method to sustain effective antitumor responses [404].

### CAR-based therapy for HCC

The mechanism of CAR-T-cell therapy relies on engineering patient T cells to specifically target tumor cells. T cells are harvested and genetically modified in vitro to express a chimeric antigen receptor (CAR), comprising an antigen-binding domain (scFv), a transmembrane anchor, and an intracellular signaling domain. This design enables CAR-T cells to bind tumor-specific antigens and promotes cytotoxic activity independent of major histocompatibility complex (MHC) presentation. Additionally, CAR-T cells can proliferate and form memory cells, providing sustained antitumor immunity. Recent advancements include second- and third-generation CARs with enhanced costimulatory domains (e.g., CD28 and 4-1BB) for improved persistence and activity. An optimal antigen for CAR-T-cell therapy in cancer should be highly expressed on tumor cells while demonstrating little to no expression on normal, healthy cells. In HCC, a significant focus is on antigens such as GPC3, which is expressed in more than 70% of HCC cases [405–408], where CAR-T cells show promising preclinical efficacy. Innovations include the use of CAR-T cells engineered to express IL-7/CCL9 [409, 410] or bispecific CARs targeting GPC3 and fibroblast activation protein (FAP) [411] to facilitate tumor migration and address tumor diversity in HCC. Similarly, CAR-T cells targeting AFP, c-Met, and NKG2DL have demonstrated tumor inhibition in preclinical settings. Other explored targets include CD133, EpCAM, MUC1, and CEA [412]. The results from ongoing clinical trials are pending deciphering whether CAR-T-cell therapy could be a new therapeutic option for HCC patients.

Despite its potential, CAR-T-cell therapy faces significant obstacles in HCC. The fibrotic TME and insufficient T-cell trafficking limit therapeutic delivery. Immunosuppressive cells, such as T_regs_ and TAMs, further hinder treatment efficacy [412, 413]. Moreover, systemic toxicity, including cytokine release syndrome (CRS) and neurotoxicity, remains a critical challenge. Innovative strategies, such as localized delivery, combination therapies with ICIs, and genetic modifications, are being explored to enhance CAR-T-cell performance while minimizing adverse effects [412].

In addition to T cells, innate immune cells such as NK cells are being explored for CAR-based therapies in other cancers and in HCC [414]. CAR-NK cells offer advantages such as a lower risk of graft-versus-host disease and the ability to target tumor cells without prior sensitization. Studies have shown that GPC3-specific CAR-NK cells can effectively target HCC cells in preclinical studies [229, 415]. In addition, chimeric antigen receptor-engineered macrophages (CAR-Ms) represent a novel and promising immunotherapeutic strategy [416]. CAR-Ms targeting tumor-specific antigens such as GPC3 and CD147 can effectively recognize and eliminate tumor cells while also secreting proinflammatory cytokines such as IFN-γ, TNF-α, and IL-12 to stimulate T-cell activation and reshape the TME [417]. These engineered cells promote an antitumor M1 phenotype and suppress the immunosuppressive M2 macrophage state, enhancing immune infiltration and tumor clearance [418]. Preclinical models have shown that combining GPC3-directed CAR-Ms enhances tumor control in small HCC lesions [418]. In addition, in preclinical models of HER2-positive solid tumors, CAR macrophages combined with anti-PD-1 therapy demonstrated strong antitumor effects. These results highlight a synergistic interaction between CAR-M cells and checkpoint inhibitors, suggesting a promising strategy to improve the response of tumors resistant to anti-PD-1 monotherapy [419].

### Conclusion and future perspectives

Despite significant advances, understanding the immune microenvironment in HCC remains a complex and evolving challenge. The introduction of immunotherapy has significantly modified the landscape of HCC management, improving patient outcomes; ICIs are currently recommended as first-line treatments for advanced HCC, and they are likely to become the standard of care for intermediate HCC when combined with locoregional therapy in the coming years. This shift encouraged us to adopt a more dynamic approach to patient management, continually reassessing the potential for curative treatments on the basis of the patient’s response. However, the immune landscape of HCC is shaped by a multitude of factors, including chronic inflammation, fibrosis, metabolic dysfunction, and gut‒liver axis dysregulation, all of which contribute to an immunosuppressive and heterogeneous TME. A major predicament lies in the dynamic and context-specific nature of immune responses, which vary not only between patients but also across disease stages and etiologies [420]. This variability hinders the identification of universal immune biomarkers and limits the predictive power of current models.

To further elucidate the intricate immune milieu in HCC, future efforts must prioritize the integration of multiomics approaches, spatial transcriptomics, and high-dimensional single-cell technologies to map immune cell phenotypes, functions, and interactions within their anatomical context [133, 421] Moreover, refined animal models that faithfully recapitulate human immune‒tumor interactions—particularly in the context of underlying liver disease—are essential for translational research. Collaborative efforts combining computational modeling, patient-derived data, and in vivo validation will be key to uncovering novel immunotherapeutic targets and improving patient stratification [422, 423] Furthermore, ongoing trials hold the promise of providing answers and will likely shape our clinical practices for the coming decades. By embracing this multilayered complexity, the field can move closer to precision immunotherapy tailored to the unique immune landscape of each HCC patient.
